# Development of GelMA-Based Hydrogel Scaffolds with Tunable Mechanical Properties for Applications in Peripheral Nerve Regeneration

**DOI:** 10.1021/acsbiomaterials.5c00023

**Published:** 2025-08-26

**Authors:** Kylie M. Schmitz, Tanner L. Larson, Michael W. Borovich, Xianfang Wu, Geyou Ao, Megan Jack, Liqun Ning

**Affiliations:** 1 Applied Biomedical Engineering Program, 2564 Cleveland State University, Cleveland, Ohio 44115, United States; 2 Department of Chemical and Biomedical Engineering, 2564 Cleveland State University, Cleveland, Ohio 44115, United States; 3 Department of Mechanical Engineering, 2564 Cleveland State University, Cleveland, Ohio 44115, United States; 4 Infection Biology Program, 22516 Lerner Research Institute, Cleveland Clinic, Cleveland, Ohio 44195, United States; 5 Department of Neurosurgery, 2569 Cleveland Clinic, Cleveland, Ohio 44195, United States; 6 Department of Neurosciences, 22516 Lerner Research Institute, Cleveland Clinic, Cleveland, Ohio 44195, United States

**Keywords:** hydrogel scaffold, viscoelasticity, single-walled carbon nanotubes (SWCNTs), 3D embedded bioprinting, electrical conductivity, human umbilical vein endothelial cells (HUVECs), Schwann cells (SCs)

## Abstract

Peripheral nerve injuries (PNIs) have a significant impact on the quality of life for patients suffering from trauma or disease. In injuries with critical nerve gaps, PN regeneration requires tissue scaffolds with appropriate physiological properties that promote cell growth and functions. Hydrogel scaffolds represent a promising platform for engineering soft tissue constructs that meet key physiological requirements. Nonetheless, ongoing innovation remains essential, as current designs continue to fall short of replicating the functional performance of autografts in bridging critical-sized nerve defects. In this study, gelatin methacrylate (gelMA)-based hydrogels are evaluated to fully characterize their pore structure, compressive stiffness, viscoelasticity, and 3D bioprintability. Hyaluronic acid (HA) and single-walled carbon nanotubes (SWCNTs) are explored as gelMA additives to modify viscoelastic and electrically conductive properties, respectively. Finally, Schwann cell (SC) and human umbilical vein endothelial cell (HUVEC) growth and functions are quantified to assess the biocompatibility of the hydrogel composites as materials for nerve scaffold fabrication. It was found that the microstructure and mechanical properties of gelMA-based hydrogels can be precisely controlled by modifying the concentrations of each component. The addition of HA led to altered viscoelastic properties of the cured structures and SWCNTs increased electrical conductivity, with both additives maintaining cytocompatibility while influencing the protein expression of both SCs and HUVECs. These composite hydrogels have potential in PNI regeneration applications.

## Graphical abstract

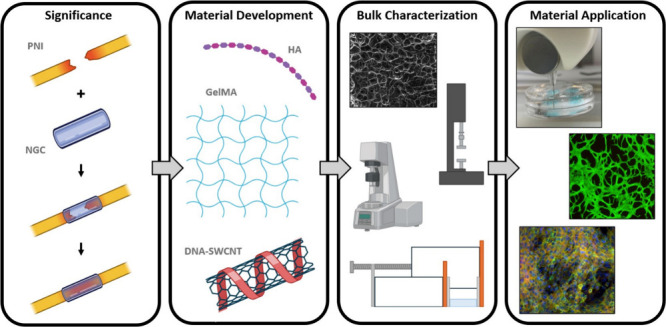

## Introduction

Peripheral nerve injuries (PNIs) due to diseases, trauma, and other accidents can have a significant impact on quality of life. Regeneration of damaged PNs with critical gaps is a major challenge, as repair requires bridging constructs. Autografts are the clinical standard for repair when damaged ends are not close enough to be directly sutured. However, due to limitations including donor site morbidity and graft availability, bioengineered scaffolds are emerging as promising alternatives. The regeneration of injured nerves relies on physiological cues that allow for the transmission of rapid neural signals. Thus, effective scaffolds must replicate bulk properties, such as mechanical stiffness of native nerve tissue, while providing a platform for effective signal transmission. As an essential step, the selection of materials for scaffold fabrication must provide a biocompatible environment to maintain the growth and functionality of neurons, Schwann cells, glial cells and endothelial cells to support neural axon regrowth and vascularization.

Hydrogels have emerged as promising biomaterials for nerve scaffold fabrication due to their biocompatibility, tunable mechanical properties, and interconnected porous networks that closely resemble the properties of the extracellular matrix (ECM) in PNs.^1,2^ In contrast to commonly used synthetic polymers such as polyesters and polyethers, hydrogels offer a tissue-like water content, rendering them mechanically and structurally more analogous to the native ECM of PNs.^3,4^ Among available hydrogels, gelatin methacrylate (gelMA) has been widely studied for tissue engineering applications.^5–8^ Compared to other used polysaccharide-based hydrogels such as alginate and chitosan,^9,10^ gelMA is a protein-based hydrogel derived from denatured collagen, which is chemically modified to be photo-cross-linkable.^5,11^ It retains natural cell-binding motifs that are critical for supporting cellular adhesion, proliferation, and other biological functions.^12,13^ Additionally, its mechanical properties can be finely tuned by adjusting the degree of methacrylation, gelMA concentration, and light exposure intensity or duration.^5,11^ While gelMA has numerous beneficial qualities for soft tissue engineering, there are opportunities for further modification to improve its cytocompatibility and comprehensive mechanical resemblance to native tissue.

Fundamental cellular processes, including growth and proliferation, are influenced by the mechanical properties of the ECM.^14,15^ When cells interact with hydrogel substrates, their behavior relies heavily on the mechanical stiffness of the material.^16^ However, the ECM in most native tissues exhibits both an immediate elastic response and a slower, time-dependent viscous response, characterized by the storage modulus (G’) and loss modulus (G”), respectively.^17–22^ Increased G” in hydrogels may change the progression of cells through mechanotransduction, causing cells to lose traction forces because of creeping or relaxation, resulting in increased cellular tension, downstream molecular functions, and enhanced cell proliferation and differentiation.^23–27^ Recent strategies focus on developing hydrogels with controlled elasticity and viscosity to promote cell growth while fulfilling functions of damaged tissue.

Modification of gelMA via conjugation with additional polymeric components can introduce an alternative mechanism for tuning viscoelasticity.^8,25,28,29^ This is likely due to the dynamic bonds between the functional groups on polymer chains within cross-linked gelMA.^17,23^ One approach toward independently tuning the viscous and elastic properties of gelMA is the addition of hyaluronic acid (HA). HA is a component of the ECM in PNs that regulates structural properties and tissue response to damage through signaling with cell receptors.^30^ HA functionality is dependent on molecular weight, distribution, concentration, cross-linking, and receptor engagement; nevertheless, its confirmed viscoelastic properties make it attractive for tissue engineering.^31^ When combined with gelMA and other biomaterials, HA improves mechanical tunability, viscoelasticity, biodegradation rate, and cell growth.^32,33^ Thus, a gelMA-HA composite provides flexibility in mechanical adjustment, allowing for the fabrication of tissue-like hydrogel substrates. However, the specific gelMA-HA interactions that govern elastic and viscous behavior have not been fully documented, necessitating further investigation for tissue repair or replacement applications.

Electrical conductivity is also critical for neural scaffold fabrication to support electrical signal transmission; however, hydrogels like gelMA and HA are natural insulators. Therefore, to improve the electrical conductivity of gelMA-based hydrogels, numerous studies have incorporated carbon nanotubes (CNTs) into the matrix.^34–37^ CNTs are visualized as rolled up graphene sheets that form cylindrical structures with nanoscale diameters, which can be functionalized via surface modifications for a variety of applications.^38,39^ They have a large aspect ratio, making them desirable for maximized adsorption,^40,41^ and can improve the electrical conductivity of hydrogels while maintaining cytocompatibility at relatively low concentrations.^42–44^ They can also impact the mechanical properties of gelMA, while promoting cell function.^45^ This concept has been explored in many cardiomyocyte-based applications, but the use of CNTs in gelMA-based hydrogels for neural applications remains underrepresented in the literature.^44,46,47^ CNTs have promoted neuronal growth and axon extension in other materials, such as chitosan/poly­(vinyl alcohol) composites.^48^ Additionally, gelMA-carbon nanofiber composites have demonstrated comprehensive mechanical and biochemical properties that are conducive to PN regeneration.^49^ These findings suggest that gelMA-CNT composites can promote neural cell functions while providing biomimetic mechanical stiffness and viscoelasticity that alternative hydrogels lack.

Most CNTs used in recent studies are multiwalled, making their composition and surface characteristics less consistent. In addition, functionalization methods are still being optimized to improve solubility, prevent cytotoxicity, and reduce adverse reactions in other organ systems.^45^ Among all available CNTs, single-walled CNTs (SWCNTs) have been explored with the goal of improving surface characteristics, due to their highly crystalline structures and batch-to-batch consistency for functionalized electrically conductive biomaterials. In some studies, SWCNTs have demonstrated enhanced biocompatibility and solubility compared to multiwalled CNTs, as they are less likely to aggregate and easier for the body to break down.^50^ However, other studies have introduced discrepancies regarding cytotoxicity, arguing that the increased aspect ratio of SWCNTs allows for higher cell interaction and decreased biocompatibility. Here, we utilized biopolymer DNA-wrapped SWCNT hybrids, since DNA coatings have been shown to improve biocompatibility for the hybrids.^51–53^ In addition, the impact of SWCNTs on the electrical conductivity and mechanical behavior of gelMA-SWCNT composites is not fully understood. Thus, the gelMA-SWCNT system must be analyzed further to determine its effectiveness in tissue engineering applications.

The goal of this study is to develop new gelMA-based hydrogels with tunable elastic, viscous, and electrically conductive properties for PN applications. We first synthesized gelMA, gelMA-HA, and gelMA-SWCNTs at varying concentrations. We then systematically investigated how additive type and concentration affect compressive stiffness, viscoelasticity, and electrical conductivity of cross-linked samples. We designed multichannel 3D scaffolds with the aim of promoting neural axon extension, then developed bioprinting methods for optimized fabrication based on rheological analysis of the uncured bioinks. Due to the temperature-dependent viscoelasticity of gelMA-based bioinks, we precisely controlled the printing temperature, pressure, and speed to ensure scaffold fidelity. Finally, we measured cell viability and functions on each hydrogel via AlamarBlue, Live/Dead, and immunostaining. Human umbilical vein endothelial cells (HUVECs) and Schwann cells (SCs) were used to confirm material cytocompatibility due to their known roles in angiogenesis, neurogenesis, axon growth, remyelination, and overall PN regeneration.^54,55^ This work demonstrates that modified gelMA-based hydrogels possess high tunability and provide a basis for constructs in PNI repair.

## Materials and Methods

### Irgacure Photocroslinking Agent (0.5% w/v) Preparation

Irgacure (0.05 g, 2-hydroxy-4’-(2-hydroxy-ethoxy)-2-methyl-propiophenone, Sigma-Aldrich 410896) was dissolved in preheated PBS (10 mL, 70 °C) in a foil-wrapped centrifuge tube for 30 min with intermittent vortexing. The Irgacure solution was then cooled to room temperature and filtered (0.22 μm), then stored at 4 °C until use.

### GelMA Synthesis

GelMA was synthesized using gelatin (gelatin from porcine skin, Sigma-Aldrich G2500) and methacrylic anhydride (MA, Sigma-Aldrich 276685). Gelatin (10 g,) was dissolved in 100 mL phosphate buffered saline (PBS, 1X) at 50 °C, while stirring rapidly (∼500 rpm) for 30 min. MA was added dropwise, then the solution continued mixing for 3 h with the top of the flask covered in parafilm to avoid evaporation. After 3 h, the solution was centrifuged, and the supernatant was added to a clean flask with 150 mL preheated (50 °C) PBS and mixed at 500 rpm for 15 min. The gelMA was transferred into dialysis bags (Spectra/Por Dialysis Tubing, Regenerated Cellulose, MWCO 12–14 kDa), which were placed into a 50 °C deionized (DI) water bath for 7 days with twice-daily water changes. The gelMA was then sterile filtered using 0.22 μm vacuum filtration units with a PES membrane. Filtered gelMA was transferred into 50 mL conical tubes and frozen overnight at −22 °C, then lyophilized for 5 days (0.2 mbar, −50 °C).

### GelMA Reconstitution

GelMA was prepared at four concentrations (6%, 8%, 10%, and 12% w/v). Irgacure (final concentration of 0.5% w/v in PBS) was preheated to 50 °C, then added to the lyophilized gelMA in a centrifuge tube wrapped in foil. The solution was placed in a 50 °C water bath to dissolve for 1–2 h with intermittent vortexing. The pH of the solution was measured and adjusted to reach 7.4.

### GelMA-HA Preparation

GelMA-HA was prepared at four concentrations of HA (0.1%, 0.2%, 0.5%, and 1% w/v; HA, Sigma-Aldrich 53747) using 8% gelMA (w/v) for each sample. Preheated, filtered Irgacure (final concentration of 0.5% w/v in PBS, 37 °C) was added to HA in a centrifuge tube covered in foil. The mixture was placed in a 37 °C water bath to dissolve for 1–2 h with intermittent vortexing. Lyophilized gelMA was then added to the HA-Irgacure mixture and placed back into the 37 °C water bath for complete dissolution, with periodic vortexing as needed. The pH of the solution was then neutralized.

### Dispersion and Purification of SWCNTs

DNA-wrapped SWCNT (i.e., DNA-SWCNT) dispersions were prepared according to a previously published procedure with modification.^56^ CoMoCAT SWCNT powder (SG65i-L39, CHASM Advanced Materials) was dispersed in 50/50 v/v% of methanol (MeOH, ≥99.8%, Fischer Scientific) and deionized (DI) water using single-stranded DNA of (GT)_20_ sequence (Integrated DNA Technologies), which is a well-known dispersant to effectively stabilize SWCNTs in aqueous and alcohol/water media.^56–58^ Specifically, a total volume of 2 mL mixture of SWCNTs:DNA = 1:2 by mass was prepared at a starting SWCNT concentration of 1 mg/mL. Subsequently, the sample was bath sonicated for 24 h at room temperature and the supernatant was collected after centrifugation at 17,000 g for 90 min at 19 °C, which was used as the stock dispersion. Solvent exchange was performed by first adding 400 μL of isopropyl alcohol (IPA, ≥99.5%, Sigma-Aldrich) into 100 μL of DNA-SWCNT sample and the mixture was centrifuged immediately afterward at 17,000 g for 2 min to obtain SWCNT precipitation. The SWCNT pellet was washed 3 times with water before redispersion in 100 μL of water by 30 min of bath sonication at room temperature. Excess, unbound DNA was removed during the solvent exchange process as well.

### Optical Spectroscopy Characterization of SWCNTs

Visible-near-infrared (vis–NIR) absorbance (400–1600 nm) measurements of DNA-SWCNT dispersions were performed on an NS3 NanoSpectralyzer (Applied NanoFluorescence, LLC) using a 10 mm path length quartz cuvette. The concentration of SWCNTs was determined using the SWCNT extinction coefficient of 41.63 mLmg^–1^cm^–1^ at 780 nm.^59^

### GelMA-SWCNT Preparation

GelMA-SWCNT samples were prepared at four concentrations of SWCNTs (0.001, 0.0025, 0.005, and 0.008 wt %) using 8% w/v gelMA for each sample. Irgacure was prepared at 70 °C (final concentration of 0.5% w/v in PBS), then cooled to room temperature, filtered, and mixed with the SWCNT dispersion to achieve each concentration. Lyophilized gelMA was then added to the mixture and placed in a 37 °C water bath to dissolve for 1–2 h with intermittent vortexing. The pH of each solution was neutralized.

### Morphological/Porosity Analysis

The microstructure, including pore size and porosity, of each hydrogel were analyzed using scanning electron microscopy (SEM). Samples were cast using SLA-printed molds, then cured via UV exposure (Uvitron 200W UVA Enhanced Lamp) for 30 s on each side to create cylindrical hydrogels with consistent dimensions (10 mm diameter, 5 mm height). Hydrogels were removed from the molds, frozen overnight, and lyophilized for 24 h (0.2 mbar, −50 °C). Samples were sputter coated with gold–palladium for 60 s before imaging, and ImageJ software was used for analysis.

### Mechanical Analysis

The mechanical properties of each sample were measured via uniaxial compression on a CellScale device. Cured cylindrical hydrogel samples were compressed at 0.025 mm/s to 50% of their initial height. The Young’s moduli were calculated using the slope of the linear region of each stress vs strain plot (0–10% strain) after regression analysis (R^2^ > 0.99). Error bars were determined using at least three replicates.

### Rheological Characterization of GelMA, GelMA-HA, and GelMA-SWCNT Samples

An Anton Paar Physica MCR 301 rotational rheometer was used to measure the viscoelastic properties of cured gelMA, gelMA-HA, and gelMA-SWCNT samples. A parallel plate geometry (25 mm diameter) was used for all experiments with a gap distance (1.0–1.4 mm) that minimizes the effect of wall slip. A Peltier temperature device hood (H-PTD200, Anton Paar) was used to control the temperature and prevent sample evaporation by placing a water-dampened Kimtech kimwipe inside. Error bars were obtained from a minimum of four replicates. Amplitude sweep measurements at an angular frequency of 10 rad s^–1^ were conducted for each sample to determine the linear viscoelastic (LVE) region at 23 °C. The critical strain corresponds to the value where a 5% decrease in the storage modulus was observed. The dynamic data was obtained within the LVE region at a strain roughly 25% less than the critical strain at 23 °C. Temperature sweep measurements of uncured 6, 8, 10, and 12% gelMA samples were conducted from 4–40 °C at an increasing rate of 2 °C min^–1^ to determine the temperature dependent behavior of storage and loss moduli at a constant strain of 5% and angular frequency of 10 rad s^–1^. The crossover point between storage and loss moduli corresponds with the melting temperature of the hydrogel, specifying constraints for bioprinting applications.

### Electrical Conductivity of GelMA-SWCNT Samples

Electrical conductivity was assessed using two-point probe resistance measurements in combination with measured dimensions of the samples. The GelMA/SWCNT samples were prepared as rectangular prisms with lengths between 15 to 20 mm, widths between 8 to 12 mm, and thicknesses between 1.5 to 3 mm. Samples were placed between two flat electrodes, composed of tin-coated copper, that were held parallel by a custom adjustable fixture. The custom setup was validated using resistors with known values. Resistance values for each sample were measured using a high resistance multimeter (Kaiweets HT118A) and recorded for approximately 14 min. Average resistance values were obtained from resistance versus time plots once steady state was reached. Bulk electrical conductivity (σ) of each sample was calculated using the distance between electrodes (*l*), measured resistance (*R*), and the cross-sectional area of the sample (*A*).

$$\sigma =\frac{l}{RA}$$

### 3D Bioprinting of GelMA-Based Multichannel Scaffolds

Computer-aided design (CAD) software (Fusion 360) was used to design a 3D scaffold with multiple channels. Bioinks were warmed to 37 °C in a water bath, pipetted into light-resistant printing cartridges, and placed into temperature-controlled printing heads, with the temperature set on the 3D bioprinter (CELLINK BIO X) based on data from rheological analysis of the uncured bioinks. Bioinks were cooled to the desired printing temperatures in the nozzles for about 30 min, then the bioprinter was calibrated, and pressure was tested. Filaments were closely observed during pressure tests to ensure that the bioinks were extruding with appropriate viscosity. Scaffolds were printed into a highly viscous 0.4% w/v Carbopol solution (Carbopol ETD 2020 polymer, Lubrizol) via embedded bioprinting. Scaffolds were then cured via UV exposure, then removed from the Carbopol bath and placed into PBS solution to rinse. Scaffolds were rinsed for at least 30 min in PBS, then channels were analyzed using dye injection.

### Cell Viability and Proliferation

Live/Dead (with calcein-AM and ethidium homodimer, Biotium) and AlamarBlue (Biotium) assays were used to determine the cytocompatibility of each hydrogel. Hydrogel samples, with varied gelMA, HA, and SWCNT compositions, were cast and cured in a TC-treated 48-well plate to create thin discs in the bottom of each well. Once polymerized, HUVECs (provided by our collaborator, Dr. Vahid Serpooshan, Emory and Georgia Tech University, originally purchased from ATCC, P18–28) were seeded onto the surface of each hydrogel at a concentration of ∼5000 cells per well. VEGF media was added (500 μL) to each well and the well plates were cultured at 37 °C with 5% CO_2_ for 2 weeks. Live/Dead and AlamarBlue assays were performed on days 1, 5, 7, and 14. An additional AlamarBlue assay was performed on day 23 to confirm the results. For Live/Dead assays, calcein-AM (1 μg/mL) and ethidium homodimer (0.5 μg/mL) were added to the cell culture medium to indicate live and dead cells, respectively. Samples were imaged using an Olympus fluorescence microscope, then cell viability was calculated using the ratio of live cells to the total cell number. For AlamarBlue assays, the AlamarBlue reagent was mixed at a 1:9 ratio in culture medium, then added to the samples for incubation. 100 μL of medium from each sample were then transferred to a 96-well plate. Absorbance was measured at 550 and 600 nm using a microplate reader (BioTek Instruments). Note that separate samples were made for each Live/Dead assay and cultured in parallel, but the same samples were reused for each AlamarBlue assay. Media (VascuLife VEGF medium complete kit, Lifeline +1% v/v P/S) was changed for all wells every 48–72 h.

### Immunocytochemistry (ICC) Staining

ICC sample preparation and staining were completed using a protocol established in our laboratory. Hydrogels were pipetted into wells of a 48 well plate and cured for 1 min. Endothelial cells (HUVECs, P18-28) and Schwann cells (SCs, RSC96 purchased from ATCC, P15-22) were seeded on the surface of each hydrogel at a density of 10,000 cells per well and cultured for 5 days with regular media changes (for HUVECs: VEGF Endothelial Complete Medium from Lifeline Cell Technology with 1% P/S; for SCs: Dulbucco’s Modified Eagle’s Medium, with 10% v/v fetal bovine serum and 1% v/v P/S, all from Sigma). Samples were fixed and stained after 5 days due to complete coverage of the hydrogel surfaces, observed by brightfield microscopy. Briefly, samples were rinsed with PBS, fixed using 10% formalin for 30 min, then permeabilized using 0.2% v/v Triton X-100 (Sigma) for 30 min. The hydrogels were blocked with 3% w/v bovine serum albumin (BSA, Sigma) in PBS for 30 min at room temperature (23 °C). Primary antibodies against CD31 and ZO-1 (HUVECs) or S100β and MPZ (SCs) (1:200 dilution each) were applied and incubated for 1 h at room temperature. Samples were rinsed three times with PBS, then secondary antibodies conjugated with Alexa Fluor 488 and Alexa Fluor 555 (1:200 dilution) were applied and incubated for 1 h at room temperature. After final PBS rinses, ProLong Gold Antifade Reagent with DAPI (Invitrogen, Waltham, MA, USA) was applied for nuclear staining. A Nikon Eclipse Ti confocal microscope (Nikon, Melville, NY, USA) was used for imaging.

### Statistical Analysis

Experimental data were presented as mean values ± standard deviation (SD). For each experiment, 3–6 sample replicates were analyzed, except in immunostaining quantification, where 30 cells were analyzed from each image. Statistical significance was assessed using one-way analysis of variance (ANOVA) with multiple comparisons, or Welch’s *t* test when only two groups were compared, conducted in GraphPad Prism. A significance level of *p* < 0.05 was considered acceptable for determining statistically meaningful differences.

## Results and Discussion

### Material Synthesis

The experimental processes consisted of material development, structural and mechanical characterization, 3D bioprinting, and cell culture and evaluation (Figure 1). GelMA synthesis has been fully developed with a matured protocol.^60^ The reaction process was thoroughly tested and refined in our lab to achieve gelMA with a precisely controlled degree of functionality (DoF). Proton NMR data confirmed that gelMA was successfully synthesized with a DoF around 60%, measured by the area under methacrylate vinyl group peaks (Figure S1). This DoF was selected to allow for ample cross-linking via methacrylate groups, while still preserving sufficient adhesive ligands to enable interactions with cells.

**Figure 1:**
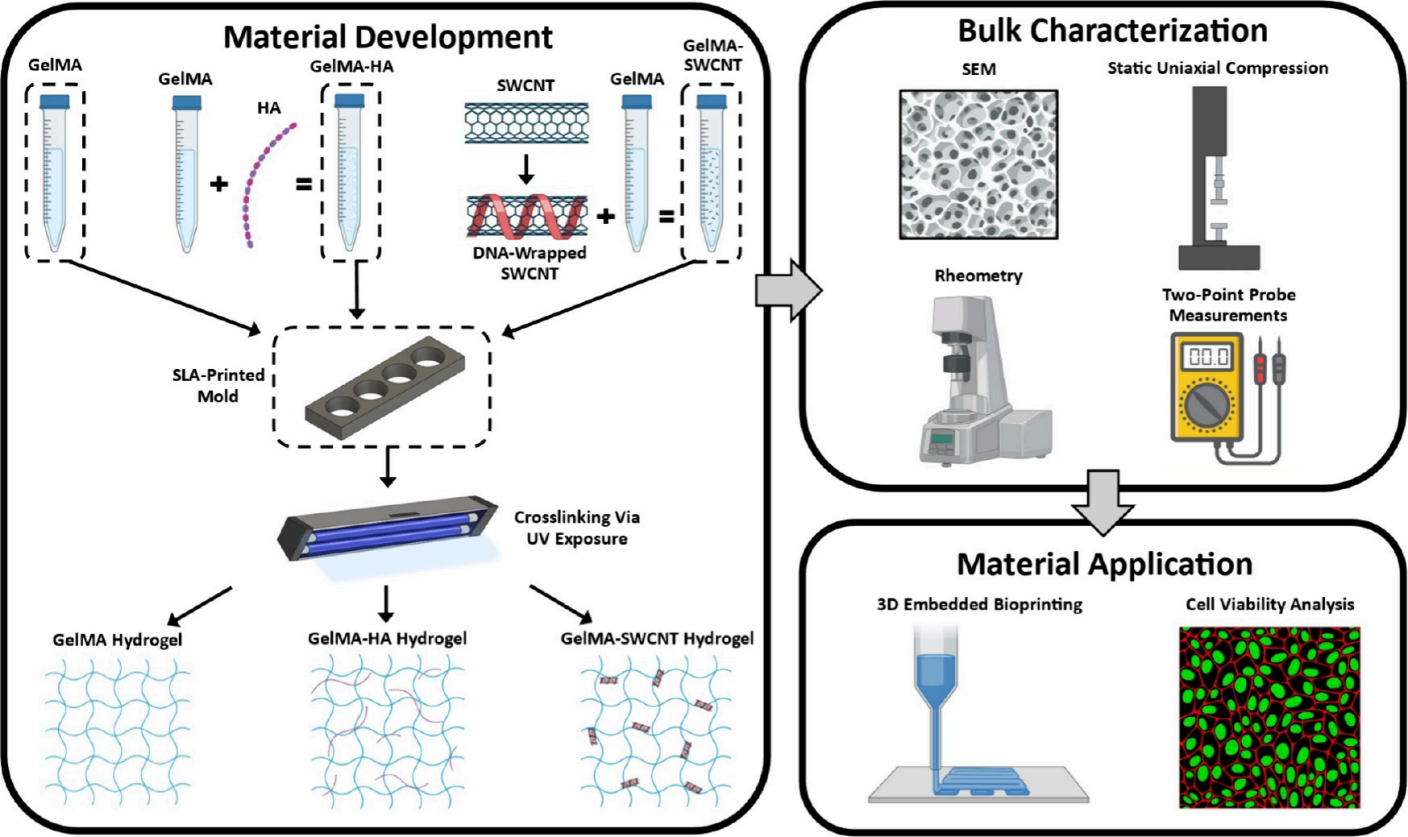
Schematic overview of material preparation, bulk characterization, and application toward 3D bioprinting and cellular work.

### Pore Size and Porosity

Hydrogels feature a microporous structure, which supports liquid retention. This structure closely mimics the ECM of natural tissue, while providing ample space for cell attachment, proliferation, and migration. Furthermore, micropores facilitate nutrient and oxygen transport, enabling cellular metabolism and promoting tissue regeneration. Therefore, precise control of pore architecture, including pore size and porosity, is crucial. The SEM images of gelMA, gelMA-HA, and gelMA-SWCNT revealed changes in pore structure between sample types, indicating controllable pore size and porosity via modification of gelMA, HA, and SWCNT concentrations (Figure 2 and Figure S2). Results from plain gelMA samples indicated decreasing pore size with increasing gelMA concentration, which aligns with observations from previous studies (Figure 2B).^61^ This demonstrates that increasing the gelMA concentration leads to the formation of a more condensed polymer network, which in turn reduces the pore size within the hydrogel.

**Figure 2:**
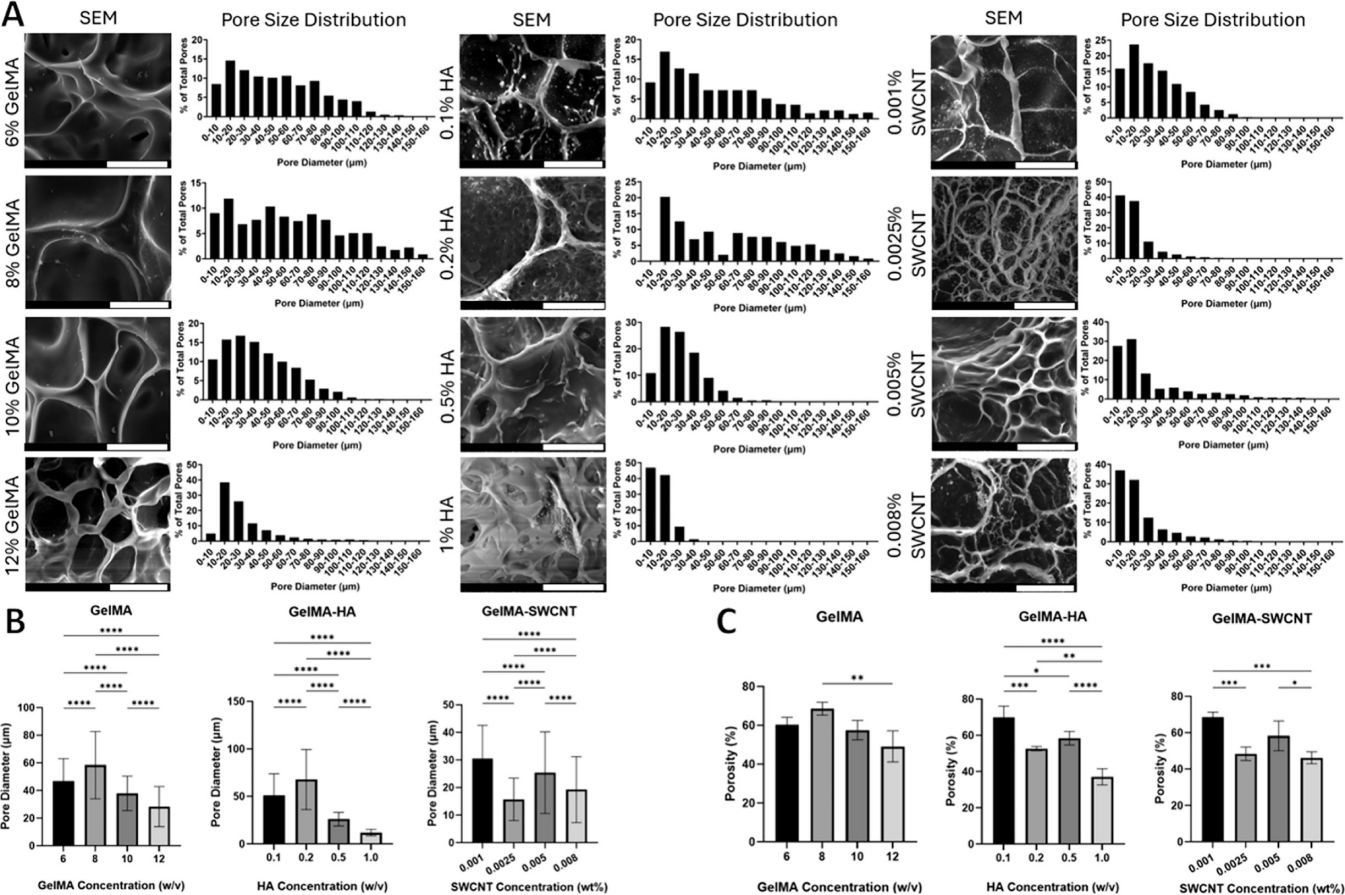
Scanning electron microscopy (SEM) images (scale bar = 50 μm) and pore size distribution of gelMA, gelMA-HA, and gelMA-SWCNT samples (A); mean pore size (B) and porosity values (C) for each cured hydrogel. * denotes *p* ≤ 0.05, ** denotes *p* ≤ 0.005, *** denotes *p* ≤ 0.001, and **** denotes *p* ≤ 0.0001; no asterisks means no significant difference; *n* = 4 images for each quantification.

Adding HA to 8% w/v gelMA led to a decreasing trend in pore size with increasing HA concentration (Figure 2). Images highlight the microlevel interactions between gelMA and HA, revealing the underlying structure of gelMA-HA composites formed through physical blending. It has been demonstrated in the literature that HA can interact with other hydrogels without a chemical cross-linker or catalyst.^62^ Fourier transform infrared (FT-IR) spectroscopy was used to confirm physical interactions between gelMA and HA that are expected to cause morphological changes in the composites (Figure S3). Specifically, it was found that the addition of >0.5% w/v HA to 8% gelMA generates a hydrogel network with decreased pore size (Figure 2B) and altered morphology (Figure 2A) compared to 8% w/v gelMA without HA. Lower concentrations of HA (0.1 and 0.2% w/v) did not cause noticeable changes in pore size or morphology, but higher concentrations of HA (0.5 and 1%), led to altered morphology and decreased pore size despite the constant gelMA concentration. Thus, controlled HA incorporation provides a practical approach to regulating the porous structures of gelMA-based hydrogels.

In gelMA-SWCNT groups, pore structure was less consistent, potentially due to nonuniform distribution of SWCNTs or the formation of aggregates.^63^ CNTs tend to form aggregates in solution due to their strong van der Waals interactions and hydrophobic surface chemistry.^64^ To mitigate this phenomenon, functionalization is widely used to establish a hydrophilic surface coating.^65^ Our SWCNT surfaces were modified using DNA to limit aggregates within the hydrogel matrix, mainly through electrostatic interactions of the charge-carrying phosphate-sugar DNA backbone on the surface of nanotubes. While the visual appearance of the cured gelMA-SWCNT hydrogels indicated thorough dispersion of SWCNTs, and an extensive mixing process was employed to evenly distribute SWCNTs, it is possible that the dispersal of SWCNTs was not perfectly uniform throughout the porous gelMA network. As a result, a range of pore sizes and porosity values was observed in each of the four gelMA-SWCNT concentrations tested. Despite these inconsistencies, a trend was still recognized with pore size and porosity decreasing as SWCNT concentration was increased. Overall, our results demonstrate that controlling gelMA, HA, and SWCNT concentrations offers an effective strategy for tuning the pore size and porosity of the resulting hydrogels.

### Compressive Stiffness

The mechanical properties of the ECM influence cellular activities and tissue progression during the regenerative process. Among these properties, stiffness, commonly measured by the elastic modulus, is a key factor and can be measured through tensile or compression tests. Here, the compressive moduli were measured using the slope of the linear region of stress–strain curves from uniaxial compression (Figure S4). Plain gelMA samples demonstrated a higher elastic modulus when the gelMA concentration was increased (Figure 3A). This aligns with previous studies, which confirm that increasing the concentration of gelMA leads to a denser polymer network, resulting in increased stiffness.^66,67^ These results also align with the SEM findings, which showed a decrease in pore size with increasing gelMA concentration (Figure 2C). Given these patterns, it can be concluded that decreased pore size, due to the densely packed polymer network, corresponds with an increased elastic modulus in gelMA-based hydrogels.

**Figure 3:**
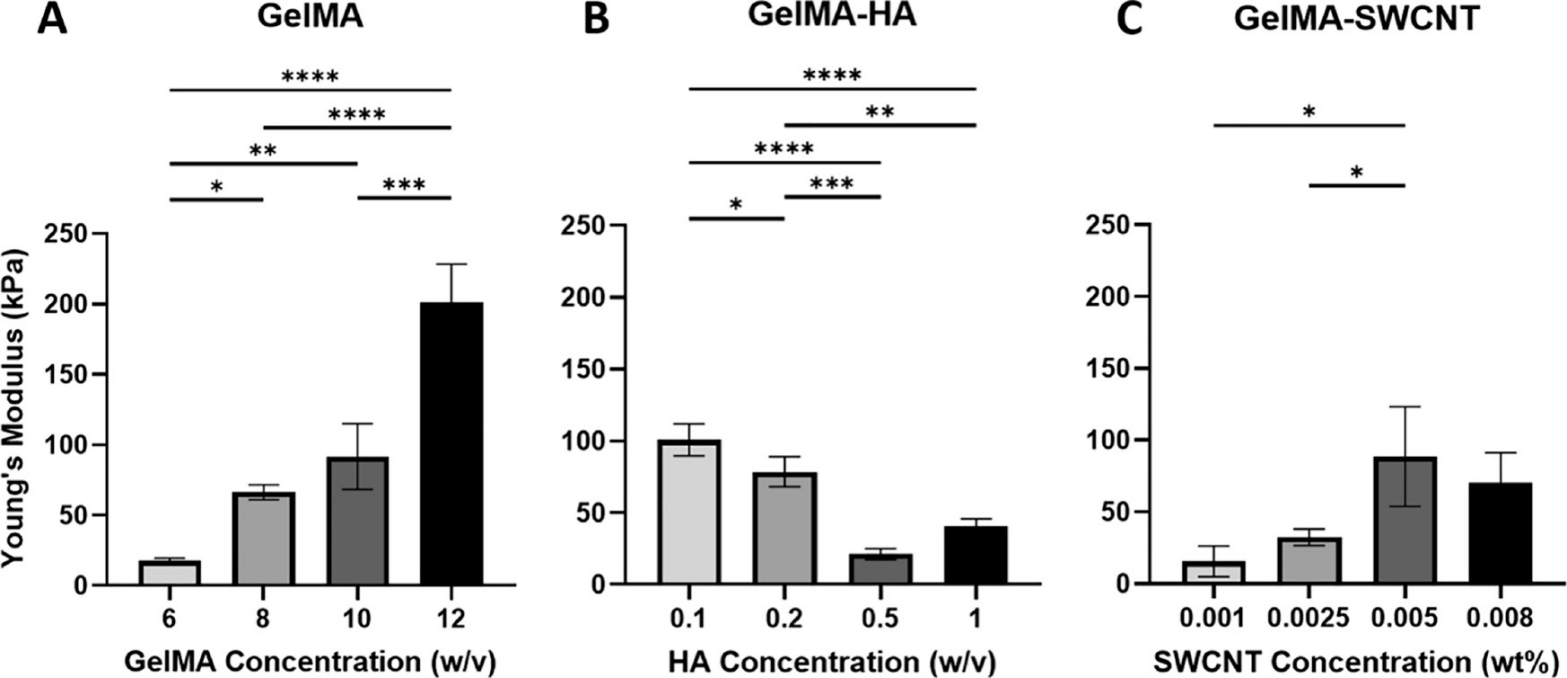
Young’s modulus of gelMA (A), gelMA-HA (B), and gelMA-SWCNT (C). * denotes *p* ≤ 0.05, ** denotes *p* ≤ 0.005, *** denotes *p* ≤ 0.001, and **** denotes *p* ≤ 0.0001; no asterisks means no significant difference; *n* = 3.

In gelMA-HA groups, samples exhibited a decreasing trend in compressive stiffness with increasing HA concentrations, except at 1% HA, where the average modulus increased compared to the 0.5% HA group, though the difference was not statistically significant (Figure 3B). In contrast, gelMA-SWCNT samples displayed increasing compressive stiffness with increasing SWCNT concentrations (no statistically significant difference between the two highest SWCNT concentrations) (Figure 3C).

The pattern observed in gelMA-SWCNT samples aligns with pore size trends in the SEM results, further demonstrating negative correlation between pore size and the bulk compressive stiffness of cross-linked hydrogels. However, gelMA-HA samples demonstrated a decrease in compressive stiffness for samples with smaller pore sizes. The opposing trends in gelMA-HA and gelMA-SWCNTs are likely due to the types of interactions between HA and gelMA compared to those between SWCNTs and gelMA. HA demonstrated physical interactions with gelMA in FT-IR data (Figure S3), which may reduce pore size through additional weak and dynamic interactions without increasing the mechanical stiffness of the hydrogel. Further studies are necessary to elucidate the bonding patterns that lead to morphological changes when HA is added at varying concentrations. Regardless, there is a clear pattern observed between SEM and compressive stiffness results that suggest tunability of pore size, porosity, and compressive stiffness via HA concentration modification.

While some studies have shown that SWCNTs can reinforce mechanical properties of hydrogels due to their intrinsic mechanical strength, mixing procedures and interactions between the base material and added nanotubes can influence the resulting material stiffness.^6^ This is observed in our results (Figure 3C), where an increase in elastic modulus was observed for 0.005 wt % SWCNT samples compared to both 0.001 and 0.0025 wt % SWCNT samples. However, as the SWCNT concentration reached 0.008 wt %, a decrease in the average mechanical stiffness was observed. While this change did not carry statistical significance, the decreasing trend indicates the possibility of aggregate formation at higher concentrations of SWCNTs, which may decrease the bulk compressive stiffness. In addition, the variety of pore sizes for each SWCNT sample group may contribute to the variations in mechanical stiffness. UV light absorption by SWCNTs may also influence the bulk stiffness of the hydrogels (Figure S5). Further studies are needed to thoroughly assess how UV absorbance by SWCNTs affects cross-linking to quantify their impact on gelMA cross-linking efficiency. A low SWCNT concentration range was selected in this study to ensure thorough mixing and minimize aggregation. Although aggregation was minimal, further optimizing SWCNT dispersion within the gelMA network could improve the consistency of compressive stiffness values and enable exploration of higher SWCNT concentrations.

### Viscoelasticity

Transmembrane ECM receptors function as mechanoreceptors, transmitting mechanical cues to the cytoskeleton through mechanotransduction to regulate cell behavior.^68^ Forces actively generated by cells must be counterbalanced by the passive forces exerted by the substrate, which are influenced by the mechanical properties of the substrates, such as elasticity. However, purely elastic substrates only store the force or energy generated by cells. This differs from natural tissues, which are viscoelastic, meaning they possess both elastic and viscous components. Viscoelasticity allows for both energy storage and time-dependent energy dissipation following matrix deformation by cells.

Rheological experiments were performed to quantify the viscoelastic properties of the gelMA-based samples. To mimic the types of forces generated by cells, dynamic tests were conducted at lower frequencies.^24^ A Peltier temperature device hood with a water-dampened kimwipe was employed to prevent curing or dehydration throughout each test (Figure S6). Rheological analysis confirmed that the mechanical properties of gelMA increase with concentration, with the G’ of cured 6%, 8%, 10%, and 12% (w/v) gelMA being about 1.5, 3.4, 6.8, and 8.9 kPa, respectively (Figure 4A). These values are similar to the storage moduli for cured gelMA found in the literature.^61^

**Figure 4:**
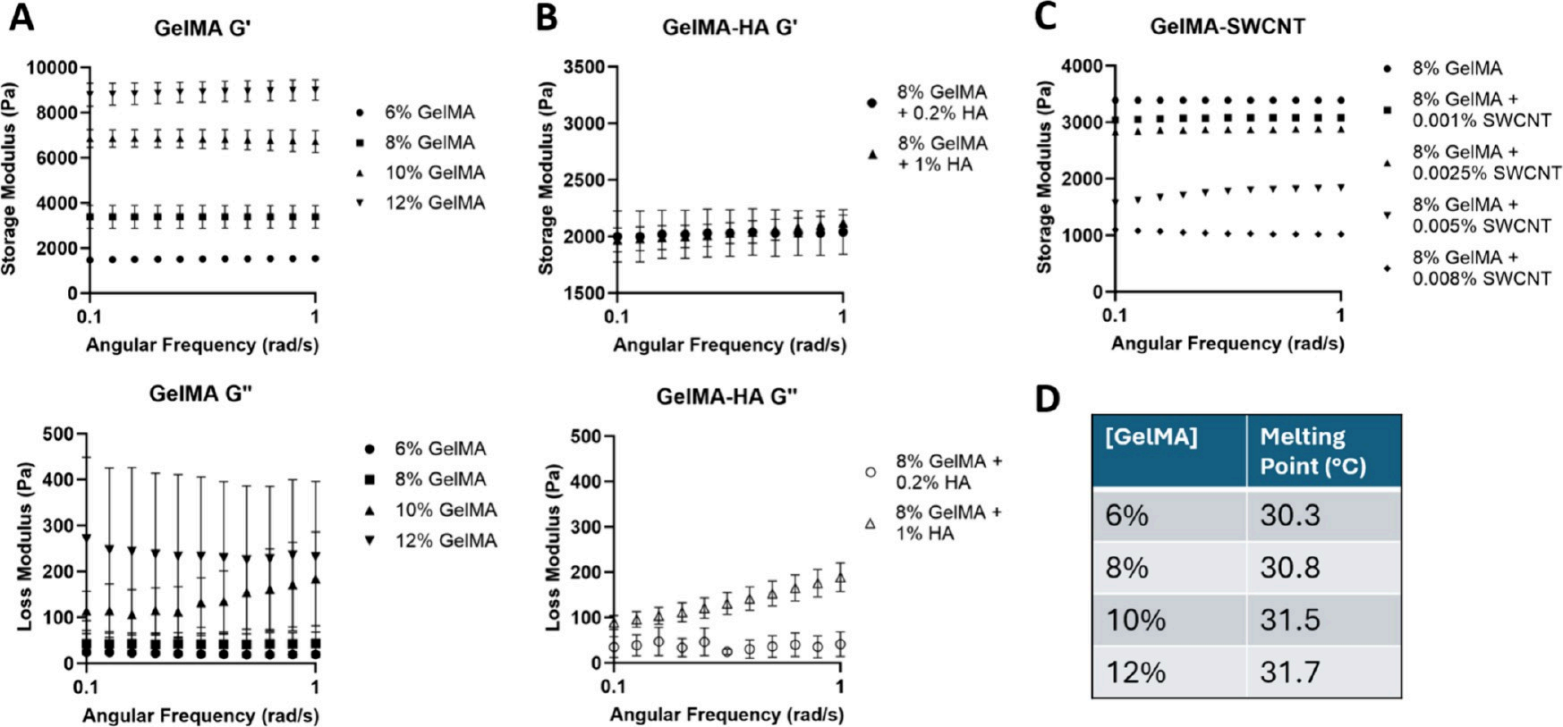
Rheological analysis of cured gelMA (A), cured gelMA-HA (B), cured gelMA-SWCNT (C), and uncured gelMA (D); *n* = 3.

The G” of gelMA samples was less consistent, compared to the G’ (Figure 4A). The G” remains sensitive to residual unreacted polymer chains and local nonhomogeneities after polymerization; thus, poor consistency in the loss modulus of cured gelMA samples was likely due to slight environmental differences in the rheological setup, particularly humidity. However, introducing HA at precise concentrations improved control over the G” of gelMA-based hydrogels, altering the G” while maintaining the G’ (Figure 4B). This suggests that uncured HA at varying concentrations can be used to independently modify the viscous characteristics of gelMA-based hydrogels while maintaining their elasticity, which is primarily impacted by gelMA concentration. One observation was that the constant storage modulus value in gelMA-HA samples was not mirrored in the compressive stiffness results for gelMA-HA, potentially due to the different types of force being applied in each test. During compression testing, a uniaxial force was applied at a constant rate, whereas rheological testing involved a dynamic oscillatory force at varying frequencies, which applied shear force rather than normal force. These results indicate that the gelMA-HA cross-linked hydrogel may respond differently to each type of stress, as well as the rate at which each type of stress was applied, since hydrogels are time-dependent materials under deformation. Further measurements for a broader range of gelMA and HA concentrations are necessary to determine the mechanism behind this response. Nevertheless, it can be concluded that G’ and G” can be tuned independently by altering the gelMA and HA concentrations, respectively. Higher gelMA concentration correlates with increased G’, while higher HA concentration correlates with increased G”. The ability to independently tune these characteristics enables future investigation of cellular responses to changes in substrate viscosity and elasticity.

GelMA-SWCNT samples exhibited a different trend, likely due to the low concentrations used and differences between the ways that HA and SWCNTs interact with gelMA. Samples with lower concentrations of SWCNTs had similar storage moduli to 8% gelMA without additives; however, the storage modulus decreased by over 1 kPa for samples with SWCNT concentrations above 0.005 wt % (Figure 4C). This is likely because low concentrations did not affect the gelMA structure enough to impact the elasticity of the hydrogel. In contrast, higher SWCNT concentrations may have caused small aggregates that decreased the elastic modulus of the hydrogel composites. In CNT/polymer composites, nonuniform dispersions and aggregation of nanotubes generally correlate to the decrease in storage modulus of composites due to deteriorated interfacial interactions.^69,70^ A difference was observed between trends for the elastic modulus of gelMA-SWCNT in compressive tests and the storage modulus of gelMA-SWCNT in rheological data. Again, this is likely due to the different types of stress being applied to the hydrogel in each test. Since the SWCNTs do not form their own entangled network, but instead can form small aggregates upon interaction with each other, they may impact the hydrogel response to these types of stress differently. As a result, higher concentrations of SWCNTs were found to increase the compressive stiffness of the hydrogel, but they decreased the elastic characteristics of the hydrogel in response to shear stress. The G” values in gelMA-SWCNT samples were variable, showing no significant differences among the sample groups. All three materials exhibited predominantly elastic behavior, with G’ values significantly higher than G”.

Temperature sweeps of uncured gelMA bioinks indicated that the phase-change temperature for 6–12% gelMA is between 30 and 32 °C (Figure 4D). The phase-change point for each uncured bioink was determined by the intersection point between the G’ and G” in temperature sweeps (Figure S7). These results were used to determine the temperature parameters for 3D bioprinting, since it is important for the printing temperature to maintain enough elasticity for mechanical support during material stacking, while possessing the necessary flow (viscous) properties for material deposition.

### 3D Embedded Bioprinting

3D bioprinting has become a useful tool in tissue engineering and regenerative medicine, revolutionizing biomanufacturing by enabling highly controlled processes to create complex, high-resolution constructs.^71^ Among bioprinting techniques, extrusion bioprinting has shown particular promise due to its ability to effectively build 3D structures through the deposition of continuous bioink filaments.^72^ However, soft materials like hydrogels can compromise structural fidelity due to spreading, fusion, and instability, which can reduce structural integrity, misguide cell growth, and prevent tissue regeneration. Embedded bioprinting offers a solution, utilizing a supporting hydrogel bath with shear-thinning properties and a specific level of yield stress. This supportive medium holds the extruded filaments in place, preventing structural distortion during the printing process and significantly improving the fidelity of 3D bioprinted constructs.

In this research, 3D embedded bioprinting was performed for gelMA (8%), gelMA-HA (8% gelMA + 0.2% HA), and gelMA-SWCNT (8% gelMA + 0.0025% SWCNT) to fabricate a rectangular model with two inner channels (Figure 5). Viscosity, gelation, and cross-linking patterns were carefully balanced to ensure that printed materials resembled the computer-aided design model while supporting cell growth.^73^ Printing pressure and speed were also controlled to maintain printability, and therefore structural fidelity, of the 3D construct.^74^ Samples were printed at temperatures between 25 and 27 °C, based on rheological evaluation, at speeds ranging from 6 to 10 mm/s to ensure that the size of the printed filament matched the size of the printing nozzle. Printing pressure was dependent on the sample type, since each sample had a different viscosity at the controlled temperature. All three gelMA-based composites were successfully printed into 3D scaffolds with high geometrical resemblance to the designed model. When dye was injected into each channel, it was clear that both channels (*d* = 1 mm) for each sample type were intact through the entire length of the scaffolds. There were no clear differences in channel structure between sample types, and the reproducibility of such models was ensured. This bioprinting model, exemplifying the capabilities of embedded bioprinting, demonstrates how its customized processes in our research enable the creation of geometrically complex structures with high structural fidelity and reproducibility. These qualities are essential as they form the foundation for use in subsequent regenerative applications.

**Figure 5:**
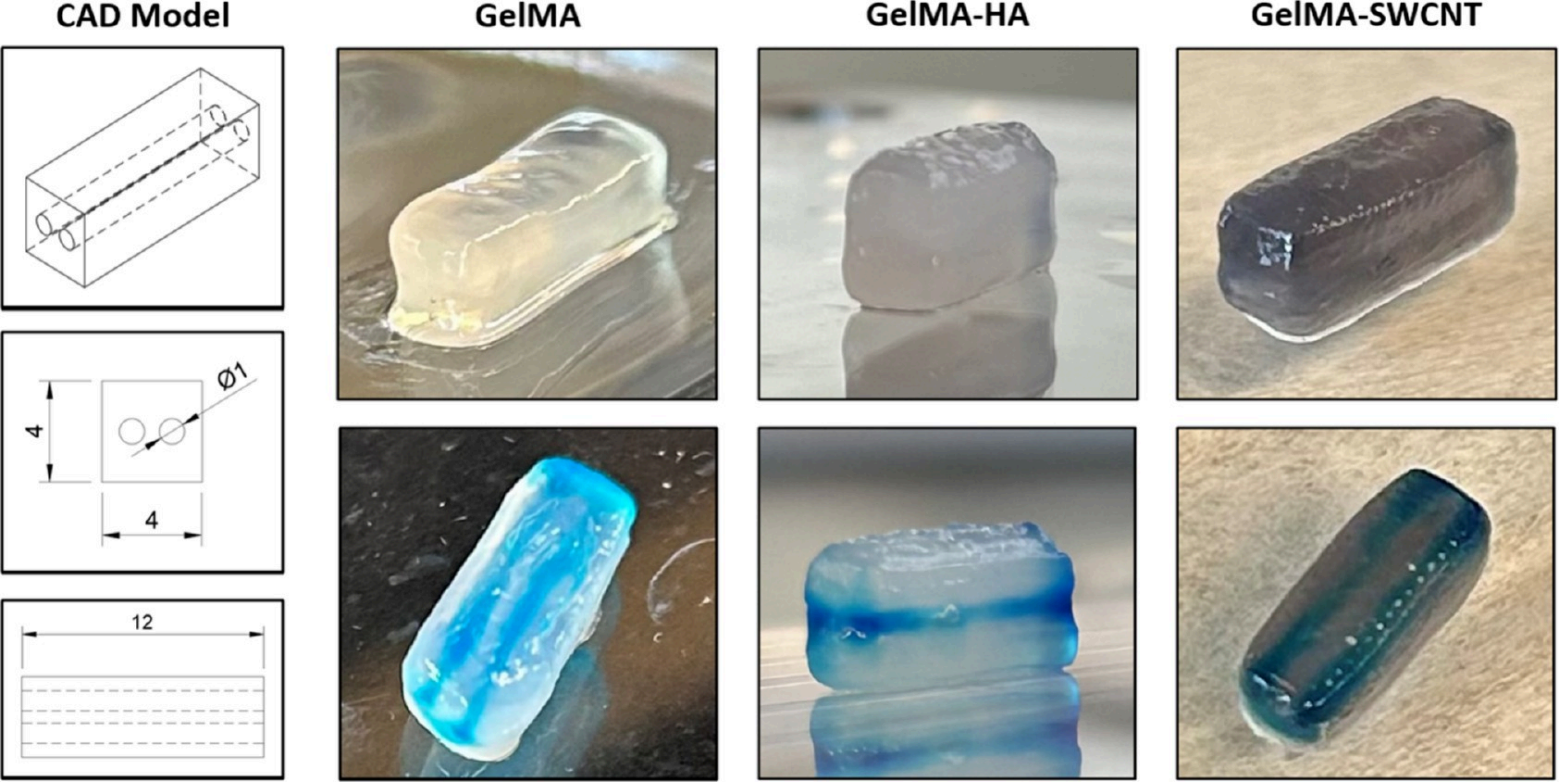
3D bioprinted rectangular constructs with multiple channels demonstrating printing fidelity of each gelMA-based hydrogel material.

### Electrical Conductivity

Electrical conductivity is a crucial characteristic for PN scaffolds, since signal transmission is critical for neural network formation and proper nerve function.^75^ It has also been shown that the growth and proliferation of electroactive cells can be promoted by electrical stimulation if a conductive environment is provided.^76^ Therefore, one of the goals of this study was to develop an electrically conductive hydrogel to allow signal transduction and provide a platform for stimulation in future experiments. Existing methods for determining the electrical conductivity of hydrogels utilize direct current (DC) resistance measurements or electrochemical impedance spectroscopy (EIS) impedance measurements. Both methods typically call for expensive, highly specialized laboratory equipment and can often require a conductive metallic paint to be applied to the sample for sufficient contact.^77–79^ Multiple iterations of the two-point probe method were explored for this study, but noticeable sample damage (color and opacity changes) and electrode corrosion were observed (Figure S8). Other challenges included difficulty with clamping the material and placing probes on the surface due to the fragile structure of hydrogels. A cost-effective device for measuring the electrical conductivity of hydrogels without damaging the samples or electrodes does not exist. Therefore, we developed a simple, yet effective, two-plate device to measure the electrical conductivity of gelMA-SWCNT samples (Figure 6A). Measurement accuracy was validated using standard alligator clips and resistors with known values (Figure 6B, see setup in Figure S9). Compared to existing methods and devices, this inexpensive setup allowed us to measure the bulk electrical conductivity of each sample without any sample damage or electrode corrosion.

**Figure 6:**
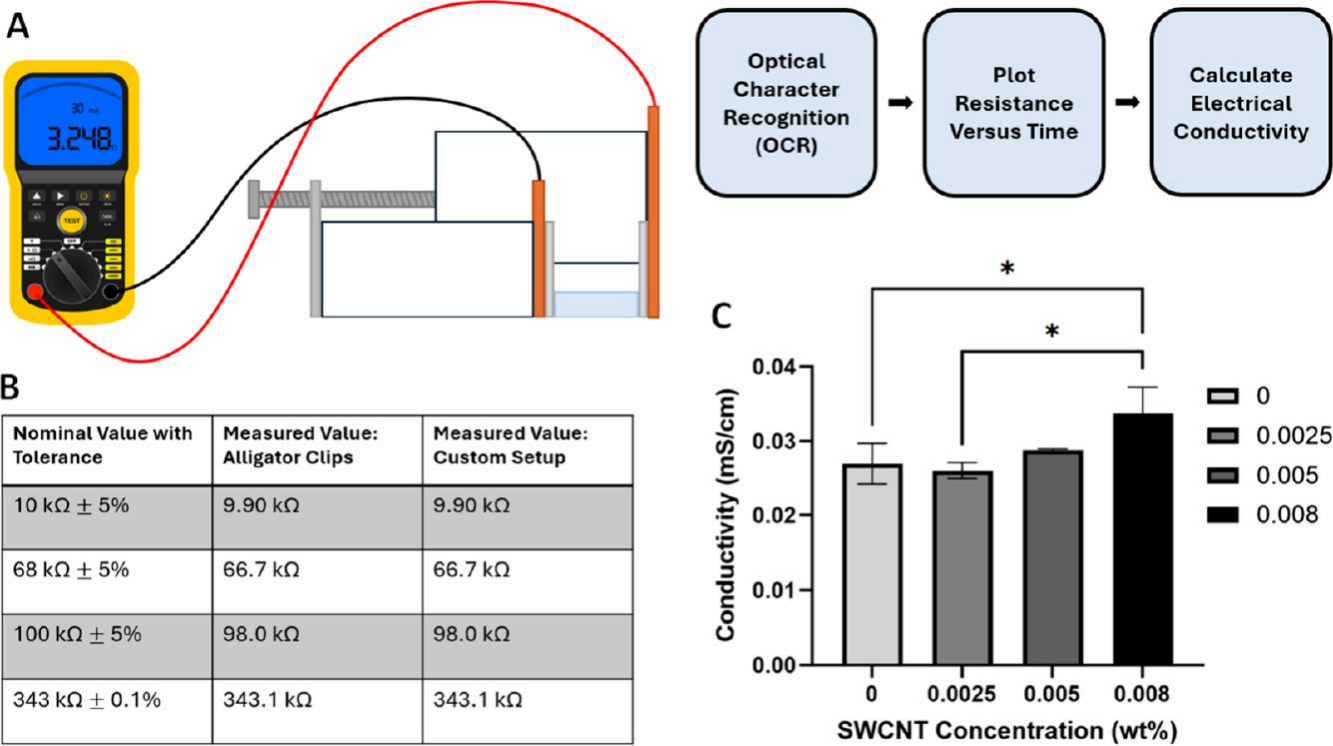
Visual diagram of electrical conductivity measurement setup and data collection process (A), validation of the novel two-plate testing device by comparison with alligator clip measurements of resistors with known values (B), and measured electrical conductivity of cured gelMA-SWCNT hydrogels, using 8% gelMA with 0.0025, 0.005, and 0.008 wt % SWCNTs (C). * denotes *p* ≤ 0.05; no asterisks means no significant difference; *n* = 3.

The apparatus developed in this study consists of a custom fixture securing two parallel tin-coated copper plates with an adjustable distance between them, allowing for measurements of samples with different geometries (Figure 6A, see setup in Figure S10). The tin-coated copper plates act as the two electrodes for measurement via the two-point probe method.^77^ Clamps were attached to each plate, ensuring complete contact with each end of the sample, while preventing any sample damage. An increasing trend was observed for the electrical conductivity of gelMA-SWCNT samples as the concentration of SWCNTs was increased (Figure 6C). A significant difference was observed between the highest concentration tested (0.008% SWCNT) and the control, as well as between the 0.008% and 0.0025% groups. The highest electrical conductivity value achieved in this study was 3.374 × 10^–5^ S/cm in the 0.008 wt % group, which is approaching the electrical conductivity of natural PN tissue (8.0 × 10^–4^ to 1.3 × 10^–2^ S/cm^80^), but remains below the physiological range of PNs. However, the marked increase in conductivity relative to plain gelMA, along with the observed concentration-dependent trend, is sufficient to investigate the influence of electrical conductivity on cellular behavior. This establishes a viable platform for next studying the effects of electrical stimulation on cells embedded in hydrogel substrates with tunable conductivity. For future refinement, further material characterization is warranted to evaluate potential SWCNT aggregation and identify stages of preparation contributing to uneven distribution. Once more consistent conductivity values are achieved, higher SWCNT concentrations may be pursued to reach the conductive properties of native nerve tissue. Despite the limitations of gelMA-SWCNT conductivity measurements, the increasing trend verifies our hypothesis and confirms findings from previous studies which state that SWCNTs can be used to improve the electrical conductivity of hydrogel-based biomaterials. In addition, our novel device allows for simple, cost-effective measurement of hydrogel conductivity without causing sample damage.

### In Vitro HUVEC Viability and Proliferation

In PN regeneration, axons are expected to have directional growth toward the distal end of the nerve gap if SCs are present. SCs guide axon growth in PNIs by migrating with them and upregulating molecules that are necessary for extension.^81^ In addition to SCs, endothelial cells promote vascularization and provide a path for SC migration, thereby facilitating nerve regeneration.^82,83^ As a representative endothelial cell type, HUVECs were initially selected in this study to evaluate the cytocompatibility of gelMA-based hydrogel substrates with modified mechanical and electrical properties.

Cell viability experiments demonstrated that gelMA, gelMA-HA, and gelMA-SWCNT are cytocompatible for HUVECs. Live/dead assays demonstrated that all hydrogel composites maintained high cell survival over 2 weeks (Figure 7A), but cell growth patterns differed between samples. Plain gelMA samples supported even distribution of cell growth. In contrast, gelMA-HA samples caused cells to grow in a more clustered pattern, with the cell density varying throughout each section of the samples. Previous work has attributed clustering patterns to the tighter and more complex pore structures in gelMA-HA substrates with high HA concentrations, which impede cell migration.^84^ Given that HA is a key ECM component in PNs that supports nerve function, its incorporation into gelMA is beneficial, but should be carefully controlled at a low level.^85^

**Figure 7:**
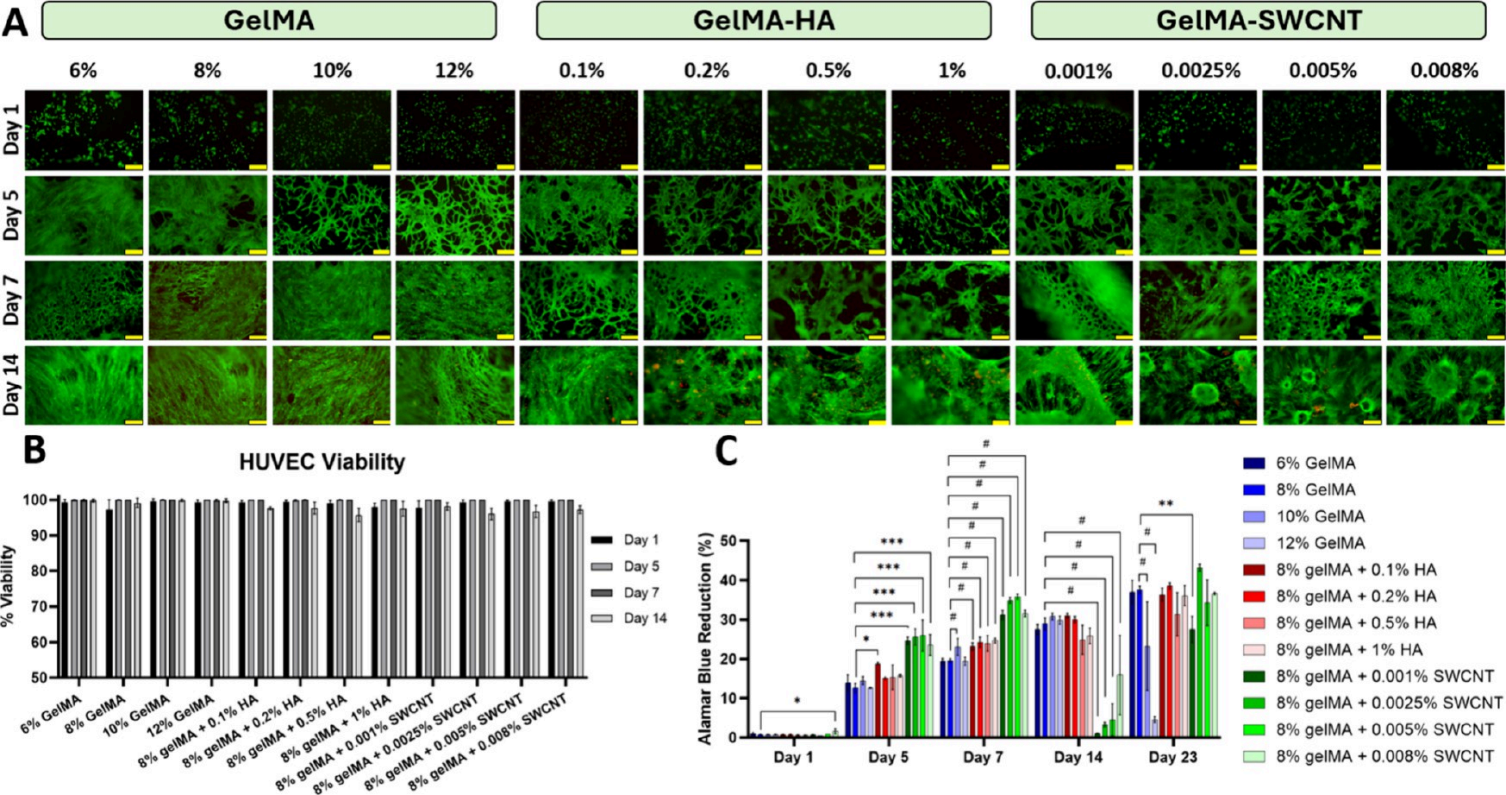
Cell viability and proliferation of HUVECs on gelMA-based hydrogel platforms, measured by Live/Dead and AlamarBlue assays (*n* = 6). Fluorescence microscopy images of HUVECs distinguished as live (green) and dead (red) on Days 1, 5, 7, and 14 (A); quantification of Live/Dead assays (B); and quantification of AlamarBlue assays, demonstrating HUVEC proliferation (C). Scale bars = 200 μm. * denotes *p* ≤ 0.05, ** denotes *p* ≤ 0.005, *** denotes *p* ≤ 0.001, and # denotes *p* ≤ 0.0001; no asterisks means no significant difference. Note that samples were compared to 8% gelMA on each day to determine statistical significance since 8% gelMA was the base material used for all additive samples.

Clustering patterns were also visible in gelMA-SWCNT samples, especially on day 14. Similar to the gelMA-HA samples, the irregular pore sizes observed in the gelMA-SWCNT samples, as shown in SEM images, may contribute to the nonuniform distribution of HUVECs. Despite these migration patterns, quantification of Live/Dead images revealed that cell viability was at least 95% for each sample throughout the 14-day period (Figure 7B). Therefore, none of the substrates were toxic to the cells, but concentrations should be controlled to allow for ample cell migration.

In addition to measuring cell growth, AlamarBlue assays were used to quantify cell proliferation (Figure 7C). Results showed that all hydrogel composites support the proliferation of cells for at least 7 days. GelMA-SWCNT samples interestingly demonstrated the highest proliferation rate compared to the other two substrates. However, day 14 measurements showed very low cell proliferation for gelMA-SWCNT samples, indicating either delayed cytotoxicity or cell death due to overcrowding within the well plate, as the proliferation rate is significantly higher than gelMA or gelMA-HA samples. To confirm that the gelMA-SWCNT materials were not cytotoxic, but that the cells simply outgrew their container due to quicker division and proliferation rates, an additional AlamarBlue assay was performed on day 23. The results demonstrated increasing cell proliferation in the gelMA-SWCNT groups again, proving that the materials were not cytotoxic to HUVECs; rather, the cells simply proliferated quickly and outgrew their container. After dead cells were removed during media changes and more room was provided for live cells to migrate, proliferation was increased again. A similar pattern was observed in the gelMA and gelMA-HA sample groups, further supporting our interpretation of the data. However, the process appeared to occur more slowly for the gelMA and gelMA-HA samples, indicating enhanced proliferation for the gelMA-SWCNT composites developed in this study. These results can be explained by our methods of SWCNT surface modification. Specific DNA strands were used to alter the surface of the SWCNTs to improve their hydrophilicity for optimized mixing. However, DNA functionalization may also have caused cells to recognize the nanoparticles more easily due to exposed functional groups similar to those on proteins and other biomolecules. Thus, the novel gelMA-SWCNT mixture not only improved mixing and prevented cell death, but it also promoted proliferation rates. Based on these findings, it can be concluded that all three sample types are cytocompatible for HUVECs over at least a three-week time period. In addition, it can be deduced that DNA surface modification of SWCNTs improves proliferation, at least for the initial stages of cell growth. Further studies should be performed to elucidate the long-term effects of DNA-wrapped SWCNTs on cells and their interactions with the regenerating nerve environment.

### ICC Staining and Quantification of HUVEC and SC Protein Expression

To further elucidate the behavior of cells on each hydrogel composite, ICC was used to quantify the expression of specific protein markers. For HUVECs, PECAM-1 (CD31) and ZO-1 were selected due to their roles in regulating angiogenic behavior, cell adhesion and migration, and tight junction formation.^86,87^ In addition, SCs were cultured on each hydrogel composite to understand the behavior of cells involved in axonal support and myelination. S100β and MPZ were analyzed in SCs due to their roles in SC function and remyelination after injury.^88,89^

In HUVEC samples, images demonstrated cell attachment and proliferation, confirming results from Live/Dead and AlamarBlue assays (Figure 8A). The addition of HA did not have any significant impact on HUVEC expression of CD31 or ZO-1 (Figure 8B), suggesting that HA (0.2% w/v) does not markedly influence the angiogenic or barrier functions of endothelial cells. We did not expand our tests to other HA concentrations, as 0.2% HA yielded the highest proliferation rate for all HA formulas (Figure 7C). However, further studies may be needed to quantify how the viscous component of the hydrogel substrate affects cellular functions at the molecular level. While initial viability and proliferation tests indicated rapid HUVEC proliferation when DNA-wrapped SWCNTs were integrated into the substrate, ICC revealed that protein expression was significantly reduced (Figure 8C). Thus, while DNA-wrapped SWCNTs may promote proliferation of HUVECs, they have an adverse effect on maturation and functionality. Future directions should further explore these trends to understand the broader and potentially multifaceted effects of SWCNTs on HUVEC behavior.

**Figure 8:**
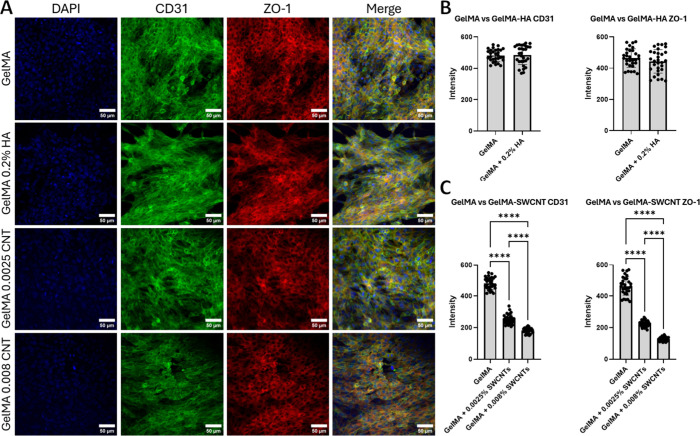
Confocal images taken after immunostaining (A), quantification of CD31 and ZO-1 expression in gelMA versus gelMA-HA samples (B), and gelMA versus gelMA-SWCNT samples (C). * denotes *p* ≤ 0.05, ** denotes *p* ≤ 0.005, *** denotes *p* ≤ 0.001, and **** denotes *p* ≤ 0.0001; no asterisks means no significant difference.

Results obtained in SC-incorporated samples shared similar trends. Images confirmed cell attachment and proliferation across the hydrogel substrates (Figure 9A). It was found that the addition of 0.2% HA led to significantly increased functions of both S100β and MPZ in SCs (Figure 9B). This is expected, as HA is a known ECM component in PNs, and its incorporation is likely to enhance interactions between SCs and the hydrogel scaffold. Identifying the optimal range of HA concentrations for PN scaffold fabrication will be an important next step. When lower concentrations of SWCNTs were added to the hydrogels, the expression of S100β did not change significantly; however, MPZ expression decreased, indicating reduced function toward myelination, and higher SWCNT concentrations reduced both markers (Figure 9C). The results consistently indicate that modified SWCNTs significantly enhance cell proliferation, potentially due to increased surface area; however, they negatively impact cellular functions, likely due to their inherent cytotoxicity even at low concentrations. Further experiments such as qPCR and Western blotting are necessary to validate these findings, and a trade-off may be necessary when determining the optimal SWCNT concentration for applications in PN regeneration.

**Figure 9:**
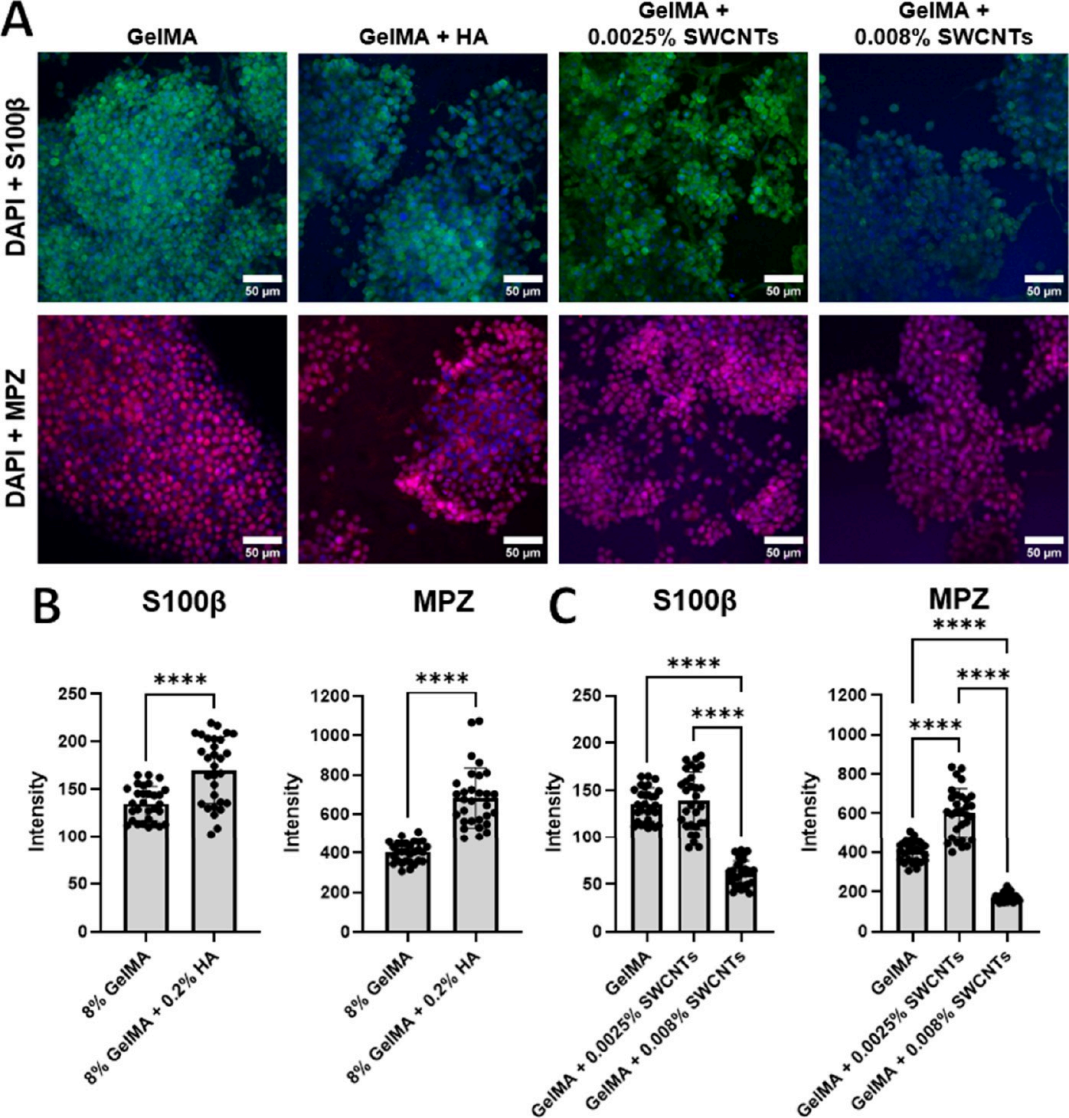
Confocal images taken after immunostaining of SCs (A), quantification of S100β and MPZ expression in gelMA versus gelMA-HA samples (B), and gelMA versus gelMA-SWCNT samples (C). * denotes *p* ≤ 0.05, ** denotes *p* ≤ 0.005, *** denotes *p* ≤ 0.001, and **** denotes *p* ≤ 0.0001; no asterisks means no significant difference.

## Conclusions

Three gelMA-based materials were synthesized with the goal of developing a tunable hydrogel capable of mimicking the PN environment for regenerative applications. The pore size and porosity of each gelMA-based composite were controllable between 10 and 100 μm and 30–80%, respectively, and the compressive stiffness was regulated between 20 and 200 kPa, all by modifying the concentrations of gelMA, HA, and SWCNTs. Rheological data showed that the storage moduli of the cured hydrogels was also tunable by composition, indicating control over the elastic characteristics of each material. When combined with gelMA, HA allowed for individual modification of the storage and loss moduli. This quality more closely resembles the properties of the ECM, which is expected, with HA being a major ECM component. Embedded 3D bioprinting, driven by the rheological properties of the prepared bioinks and printing optimization, successfully produced gelMA-based multichannel scaffolds with structural fidelity. A novel electrical conductivity testing device was developed to measure fragile materials, such as hydrogels. Conductivity measurements using this device showed an upward trend with increased SWCNT concentration. Finally, Live/Dead and AlamarBlue assays proved that all three hydrogel composites promote HUVEC growth and proliferation over a 14-day period. The surface modification of SWCNTs with DNA led to enhanced cell proliferation in the gelMA-SWCNT groups. Despite this, SWCNTs led to decreased protein expression in both HUVECs (CD31 and ZO-1) and SCs (S100β and MPZ). The reduction in expression indicates reduced support toward angiogenesis, cell adhesion, and migration in HUVECs, as well as SC function and remyelination.

Although the electrical conductivity values of the hydrogels in this study were significantly elevated, they did not reach those observed in natural PN tissue. Increased SWCNT concentrations or alternative hydrogels with intrinsic conductive properties could be explored to address this limitation.^90^ Our future work envisions utilizing purified SWCNTs that are highly enriched in metallic nanotubes, allowing tuning of the scaffold conductivity to a significantly greater extent.^57,58^ Future work should also evaluate the long-term effects of DNA-wrapped SWCNTs on cells in regenerating tissue. Biological functions of SCs and HUVECs should be more thoroughly evaluated using ELISA, PCR, etc. in future studies to fully understand molecular responses to material properties. Despite these limitations, it was concluded that the comprehensive mechanical properties, pore structure, electrical conductivity, and printability of gelMA-based hydrogels can be effectively tuned using HA and SWCNTs, while maintaining cytocompatibility. The resulting composites have potential as scaffolds in critical gap PNI repair.

## Supplementary Material



## Notes

(1) H NMR data showing a successful gelMA synthesis, (2) additional SEM figures, including an image of the sample setup, (3) FT-IR analysis of gelMA versus gelMA-HA samples and regions of interest, (4) complete stress versus strain curves for compressive stiffness experiments that were used to determine the Young’s modulus, (5) a comparison between the UV cure box spectral distribution and SWCNT absorbance spectra showing overlap, (6) rheological sample preparation and system setup, (7) temperature sweeps (rheometry) of uncured gelMA showing phase-change temperatures, (8) sample damage and electrode corrosion caused by each unsuccessful electrical conductivity measurement method, (9) validation of the custom electrical conductivity setup, and (10) labeled diagram of the custom electrical conductivity measurement device (PDF)
