# Supplementary material for: Development of GelMA-Based Hydrogel Scaffolds with Tunable Mechanical Properties for Applications in Peripheral Nerve Regeneration
Source: ACS Biomater Sci Eng. 2025 Aug 26;11(9):5467–81. doi: 10.1021/acsbiomaterials.5c00023 (PMC12421523; doi:10.1021/acsbiomaterials.5c00023)
Supplement: Supplementary file 1 [file ab5c00023_si_001.pdf]

Supporting Information

Development of GelMA-based Hydrogel Scaffolds with Tunable Mechanical Properties for Applications in Peripheral Nerve Regeneration

Kylie M. Schmitz[1,2]; Tanner L. Larson[2]; Michael W. Borovich[3]; Xianfang Wu[4]; Geyou Ao[2]; Megan Jack[5,6]; Liqun Ning[3,*]

[1]Applied Biomedical Engineering Program, Cleveland State University, Cleveland, Ohio 44115, United States
[2]Department of Chemical and Biomedical Engineering, Cleveland State University, Cleveland, Ohio 44115, United States
[3]Department of Mechanical Engineering, Cleveland State University, Cleveland, Ohio 44115, United States
[4]Infection Biology Program, Lerner Research Institute, Cleveland Clinic, Cleveland, Ohio 44195, United States
[5]Department of Neurosurgery, Cleveland Clinic, Cleveland, Ohio 44195, United States
[6]Department of Neurosciences, Lerner Research Institute, Cleveland Clinic, Cleveland, Ohio 44195, United States
*Corresponding author: l.ning@csuohio.edu

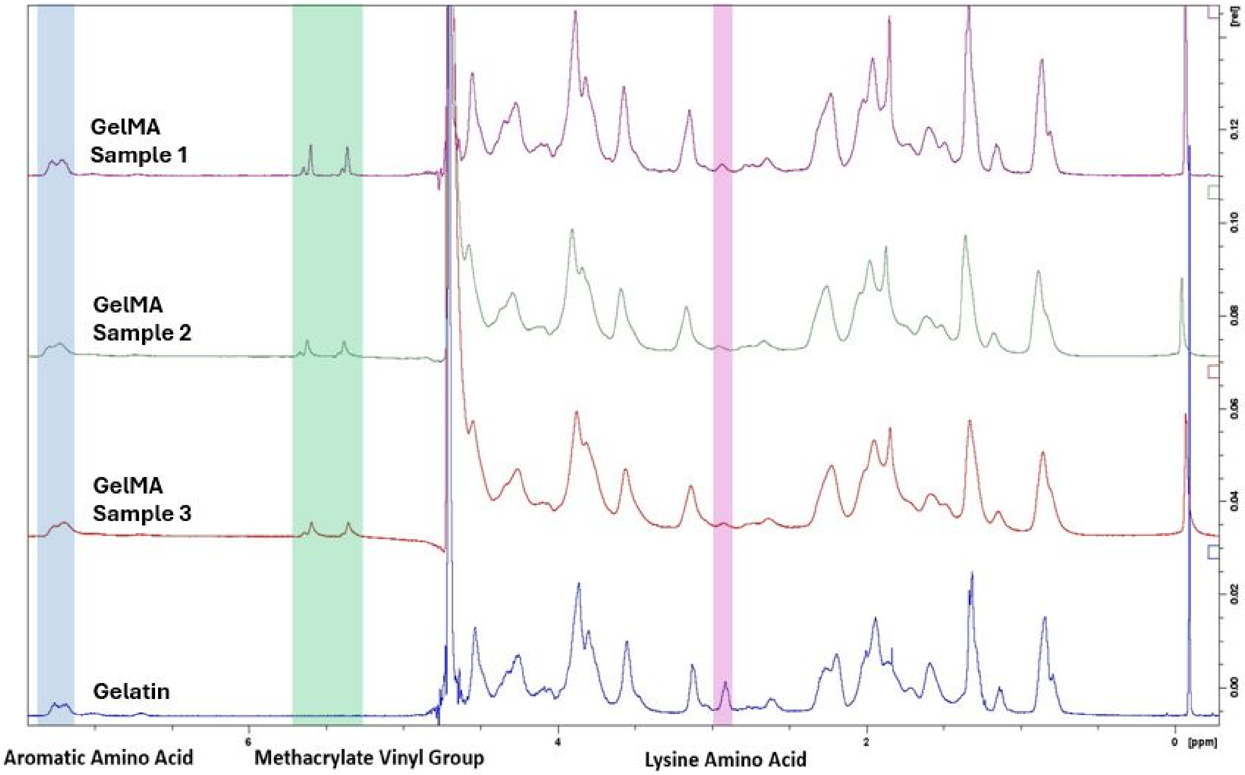

Figure S1: Proton NMR spectra of gelMA samples compared to gelatin, highlighting regions that indicate gelatin methacrylation.

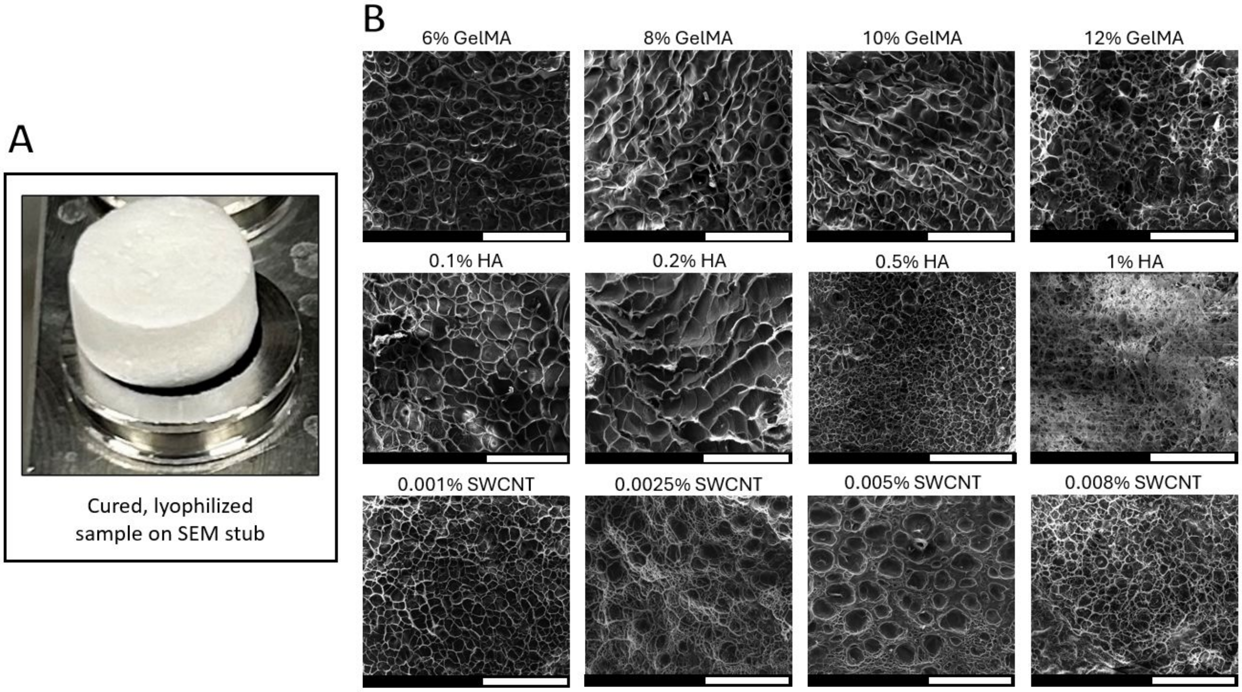

Figure S2: Cured, lyophilized hydrogel sample on SEM stub (A); SEM images of each hydrogel at low magnification for broader view (B); Scale bars = 500 μm**.**

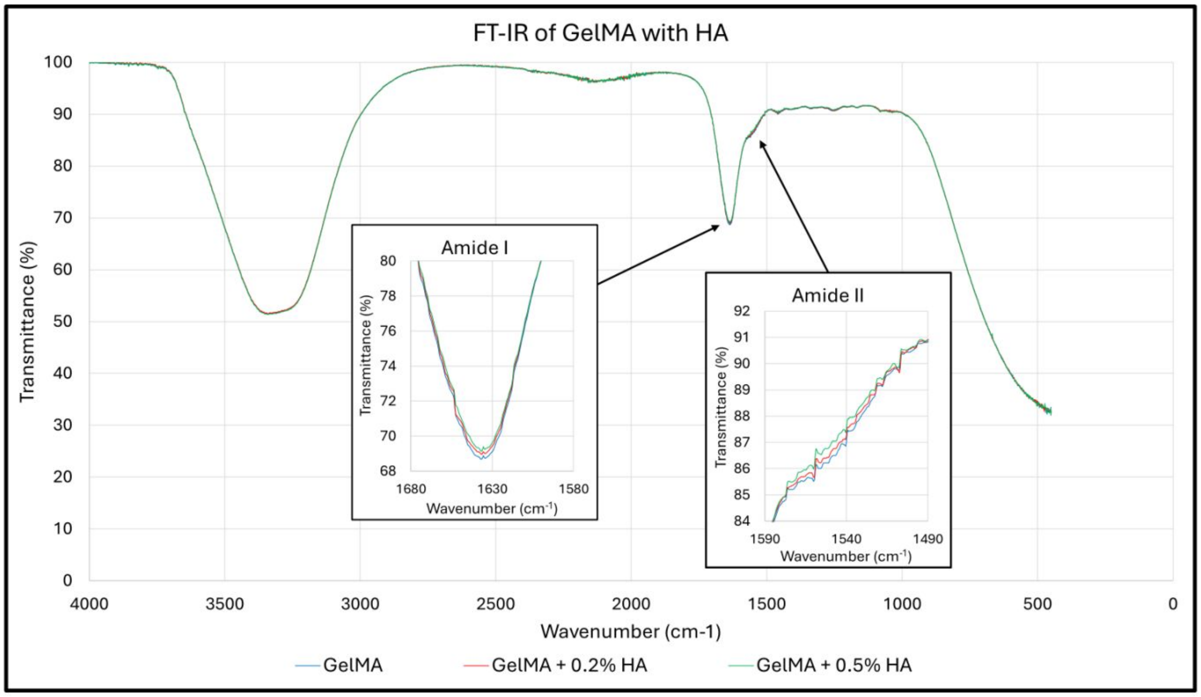

Figure S3: Fourier transform infrared (FT-IR) spectra of gelMA, compared to gelMA with different concentrations of HA. Amide I and amide II peaks are enlarged to indicate physical bonding between gelMA and HA.

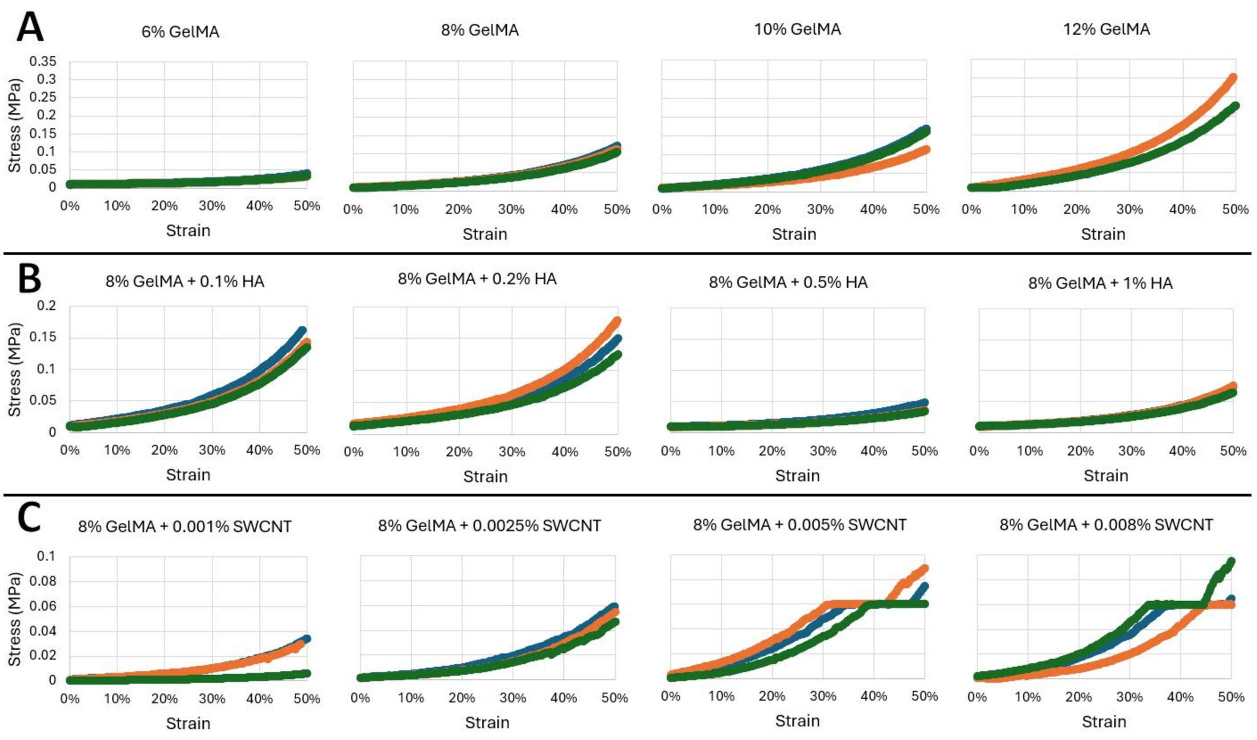

Figure S4: Compressive stiffness of gelMA (A), gelMA-HA (B), and gelMA-SWCNT (C), represented as stress vs strain curves. The three curves on each plot represent the three replicate samples.

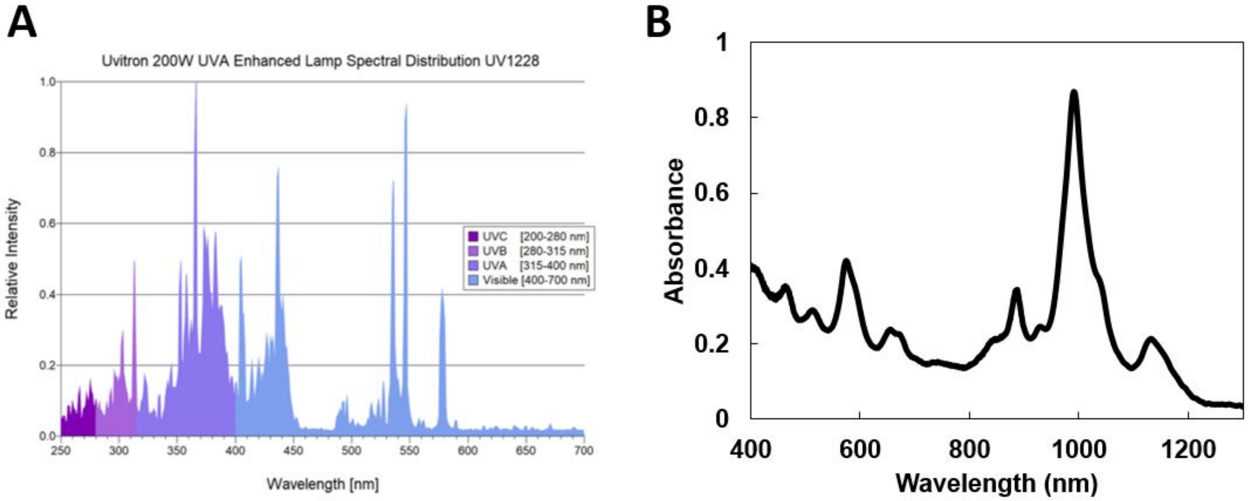

Figure S5: Spectral distribution of Uvitron 200W UV cure box (A) and absorbance spectra of SWCNTs (B), demonstrating overlap that may lead to curing effects. For panel B: purified DNA-SWCNT absorbance spectra diluted 300x in DI water; concentration of sample determined by extinction coefficient at 780 nm.

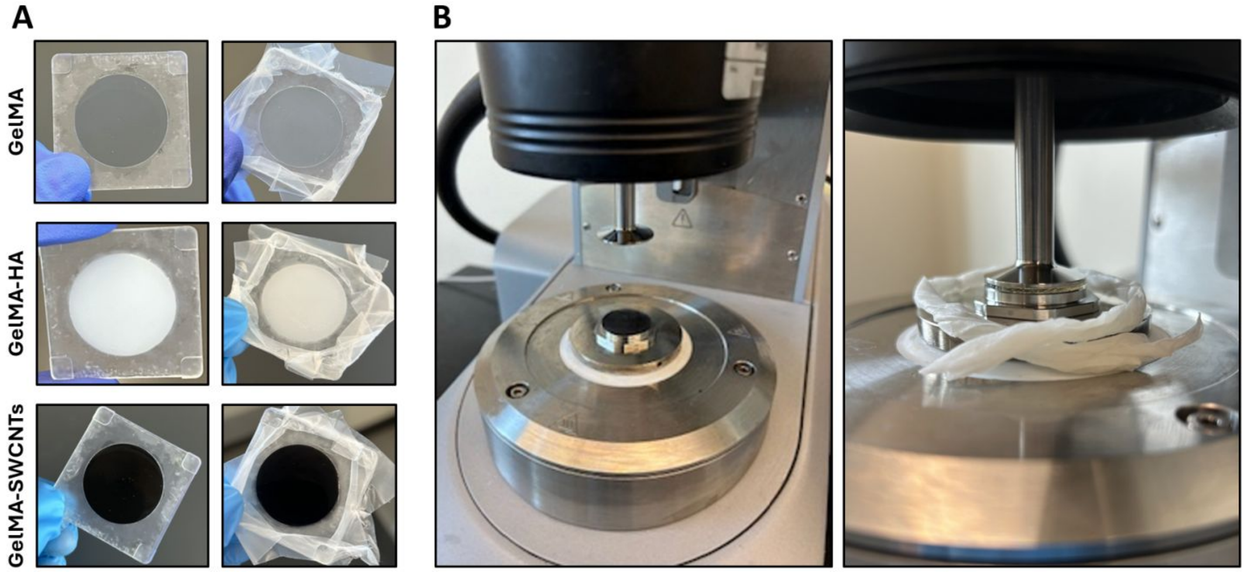

Figure S6: Rheological sample preparation and measurement system setup. Panel A displays color and opacity changes that were observed after crosslinking in samples with HA or SWCNTs. Panel B depicts modifications made to minimize the effects of light exposure and humidity fluctuations during rheological measurements (involving a Peltier hood with a water-dampened Kim wipe).

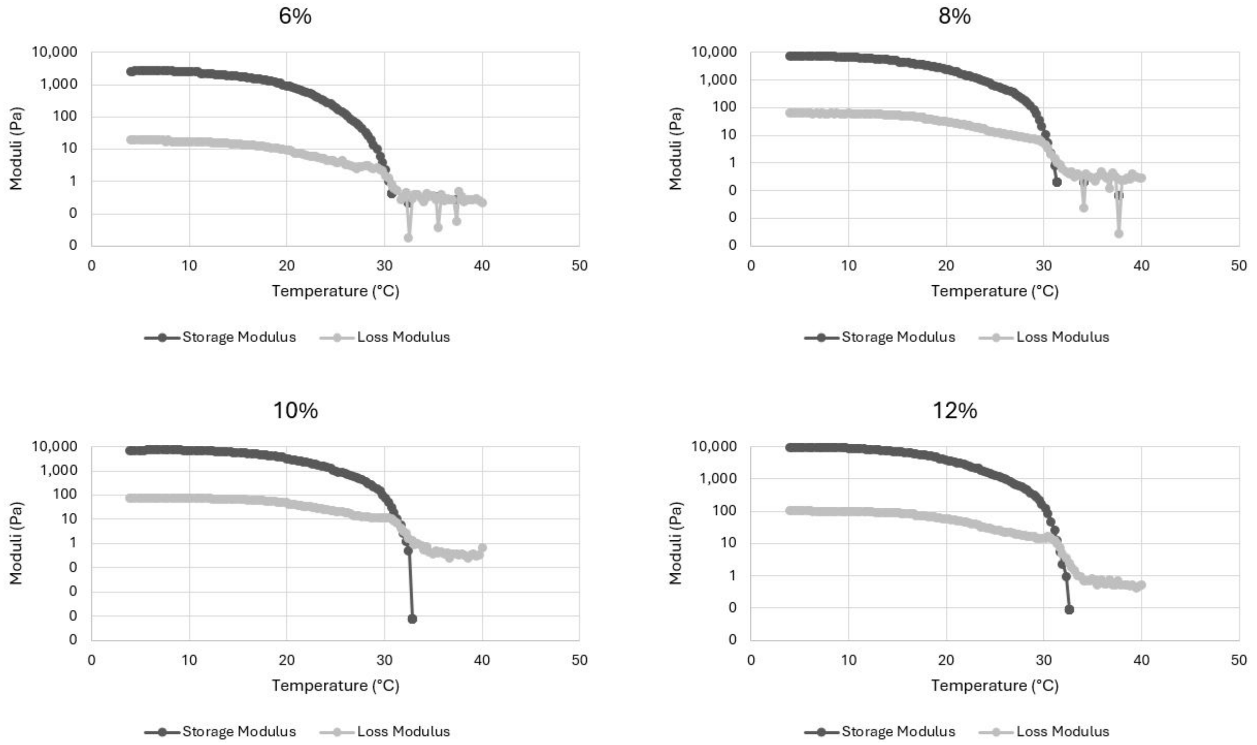

Figure S7: Temperature sweeps of uncured 6, 8, 10, and 12% gelMA, indicating the phase change point at the intersection between storage and loss moduli.

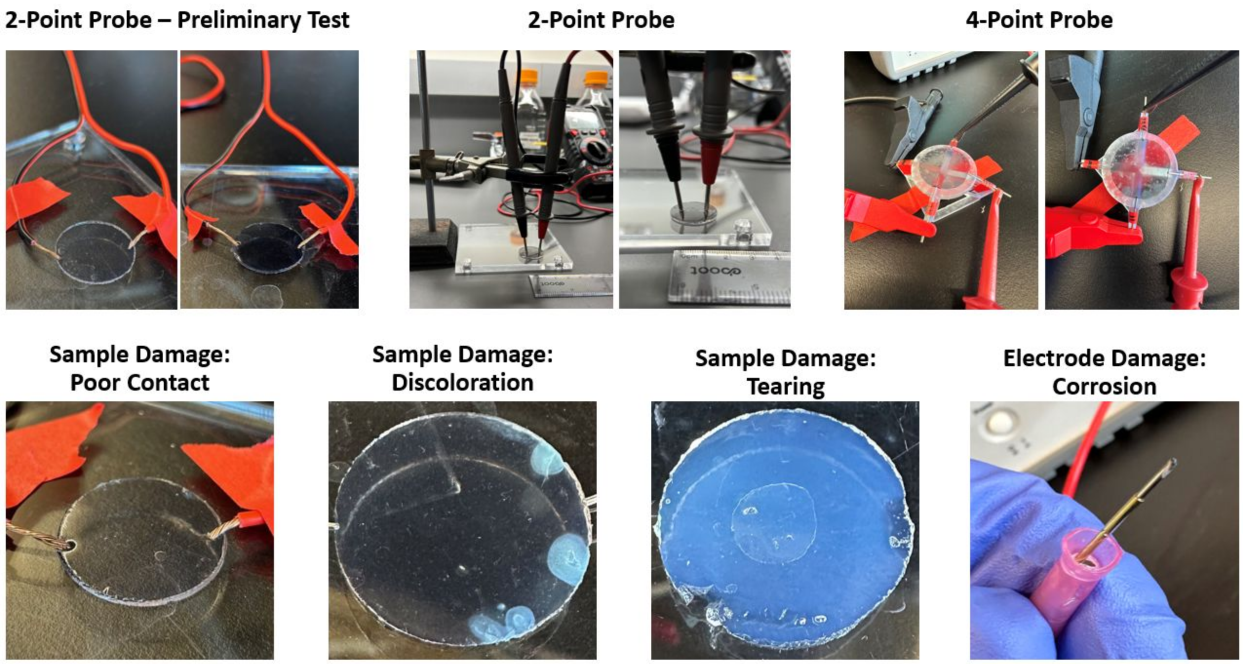

Figure S8: Attempted conductivity measurements via 2-point and 4-point probe methods showing resulting sample damage and electrode corrosion.

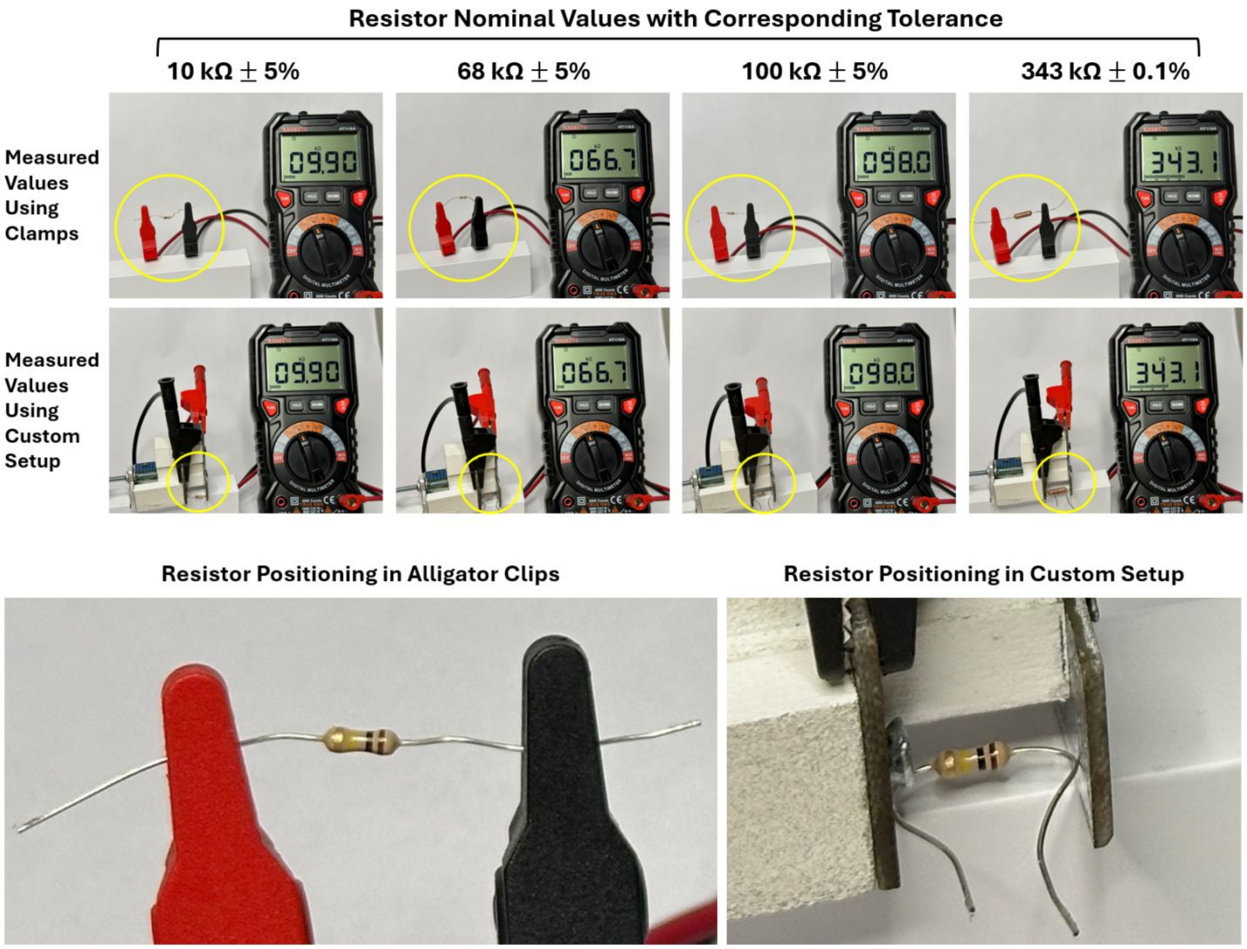

Figure S9: Validation of the custom conductivity testing setup, including nominal resistor values, tolerances, and measurements using standard alligator clips for comparison. Resistor positions for each setup are highlighted in yellow circles and enlarged for clarity.

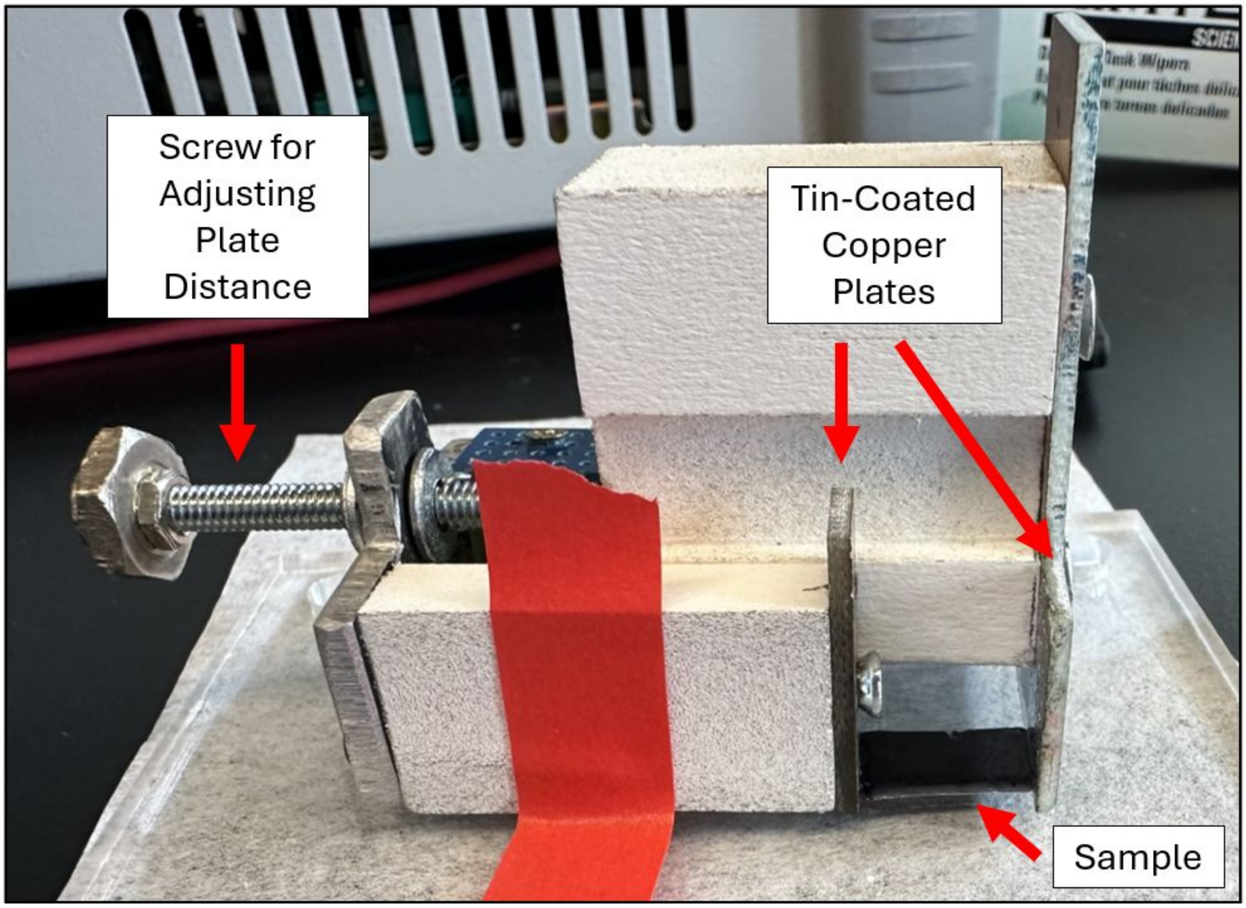

Figure S10: Custom built electrical conductivity testing device setup showing adjustable fixtures and sample placement.
